# Enolase of *Streptococcus suis* serotype 2 promotes biomolecular condensation of ribosomal protein SA for HBMECs apoptosis

**DOI:** 10.1186/s12915-024-01835-y

**Published:** 2024-02-08

**Authors:** Hexiang Jiang, Yi Sun, Fengyang Li, Xibing Yu, Siyu Lei, Sulan Du, Tong Wu, Xuan Jiang, Junhui Zhu, Jun Wang, Yalu Ji, Na Li, Xin Feng, Jingmin Gu, Wenyu Han, Lei Zeng, Liancheng Lei

**Affiliations:** 1 State Key Laboratory for Diagnosis and Treatment of Severe Zoonotic Infectious Diseases, Key Laboratory for Zoonosis Research of the Ministry of Education, Institute of Zoonosis, and College of Veterinary Medicine, Jilin University, Changchun, 130062 China; 2 Department of Respiratory Medicine, Center for Infectious Diseases and Pathogen Biology, Key Laboratory of Organ Regeneration and Transplantation of the Ministry of Education, State Key Laboratory for Diagnosis and Treatment of Severe Zoonotic Infectious Diseases, The First Hospital of Jilin University, Changchun, 130021 China; 3 Department of Veterinary Medicine, College of Animal Science, Yangtze University, Jingzhou, 434023 China; 4 Bethune Institute of Epigenetic Medicine, The First Hospital of Jilin University, Changchun, Jilin China; 5 International Center of Future Science, Jillin University, Changchun, Jilin China

**Keywords:** *Streptococcus suis* serotype 2, Enolase, Ribosomal protein SA, Liquid–liquid phase separation, Apoptosis

## Abstract

**Background:**

Ribosomal protein SA (RPSA) of human brain microvascular endothelial cells (HBMECs) can transfer from the cytosol to the cell surface and act as a receptor for some pathogens, including *Streptococcus suis* serotype 2 (SS2), a zoonotic pathogen causing meningitis in pigs and humans. We previously reported that SS2 virulence factor enolase (ENO) binds to RPSA on the cell surface of HBMECs and induces apoptosis. However, the mechanism that activates RPSA translocation to the cell surface and induces ENO-mediated HBMEC apoptosis is unclear.

**Results:**

Here, we show that RPSA localization and condensation on the host cell surface depend on its internally disordered region (IDR). ENO binds to the IDR of RPSA and promotes its interaction with RPSA and vimentin (VIM), which is significantly suppressed after 1,6-Hexanediol (1,6-Hex, a widely used tool to disrupt phase separation) treatment, indicating that ENO incorporation and thus the concentration of RPSA/VIM complexes via co-condensation. Furthermore, increasing intracellular calcium ions (Ca^2+^) in response to SS2 infection further facilitates the liquid-like condensation of RPSA and aggravates ENO-induced HBMEC cell apoptosis.

**Conclusions:**

Together, our study provides a previously underappreciated molecular mechanism illuminating that ENO-induced RPSA condensation activates the migration of RPSA to the bacterial cell surface and stimulates SS2-infected HBMEC death and, potentially, disease progression. This study offers a fresh avenue for investigation into the mechanism by which other harmful bacteria infect hosts via cell surfaces’ RPSA.

**Supplementary Information:**

The online version contains supplementary material available at 10.1186/s12915-024-01835-y.

## Background

*Streptococcus suis* serotype 2 (SS2) is an emerging zoonotic pathogen that can cross the host blood–brain barrier (BBB) and invade the central nervous system, causing meningitis in pigs and humans [1]. Ribosomal protein SA (RPSA), also known as laminin receptor 1 (LAMR1), is a multifunctional protein mainly localized in the nucleus, cytoplasm, endoplasmic reticulum, Golgi apparatus, cell membrane and extracellular vesicles [2, 3]. Various pathogenic microorganisms cross the BBB via an RPSA-mediated process [4]. We previously reported that SS2 virulence factor enolase (ENO) binds to RPSA on the cell surface of brain microvascular endothelial cells (BMECs) and induces apoptosis and destruction of the BBB [3, 5]. Therefore, RPSA is a promising therapeutic target for treating nervous system infections [4, 6].

RPSA contains two main parts, the N-terminal part with an RPS2-like globular domain and the C-terminal part with an internally disordered region (IDR) [7]. Notably, the C-terminal IDR of RPSA is an extracellular domain for interaction with 18S rRNA, immunoglobulins, and laminin 1, among others [8]. It turns out that the 263 amino acid (AA)-282 AA region is also a flexible platform for virulence factor interaction during *Streptococcus pneumoniae*, *Neisseria meningitidis*, *Haemophilus influenzae*, and Sindbis virus infection of host cells [6]. However, the mechanism that activates RPSA translocation to the cell surface is unknown.

Recent studies have reported that proteins with IDRs can undergo liquid–liquid phase separation (LLPS) to drive the formation of biomolecular condensates [9]. These LLPS condensates have multiple roles in pathogenic microbial infection, such as small-molecule drugs that suppress LLPS may become a potential strategy for the treatment of SARS-CoV-2 infection [10]. In this study, we found that stimulation of BMECs by ENO promoted the translocation of RPSA from the intracellular environment to the membrane in an RPSA-IDR-dependent manner. ENO promoted the liquid-like condensation of RPSA and enhanced its incorporation and concentration with RPSA and vimentin (VIM), which induced cell apoptosis. Furthermore, ENO stimulation elevated the intracellular level of calcium ions and increased cytotoxic activity against HBMEC cells. Our findings illustrate the important role of RPSA in the process of SS2 infection and indicate RPSA as an attractive target for the treatment of SS2 infection.

## Results

### IDR drives RPSA to form liquid condensates and translocation

**Fig. 1:**
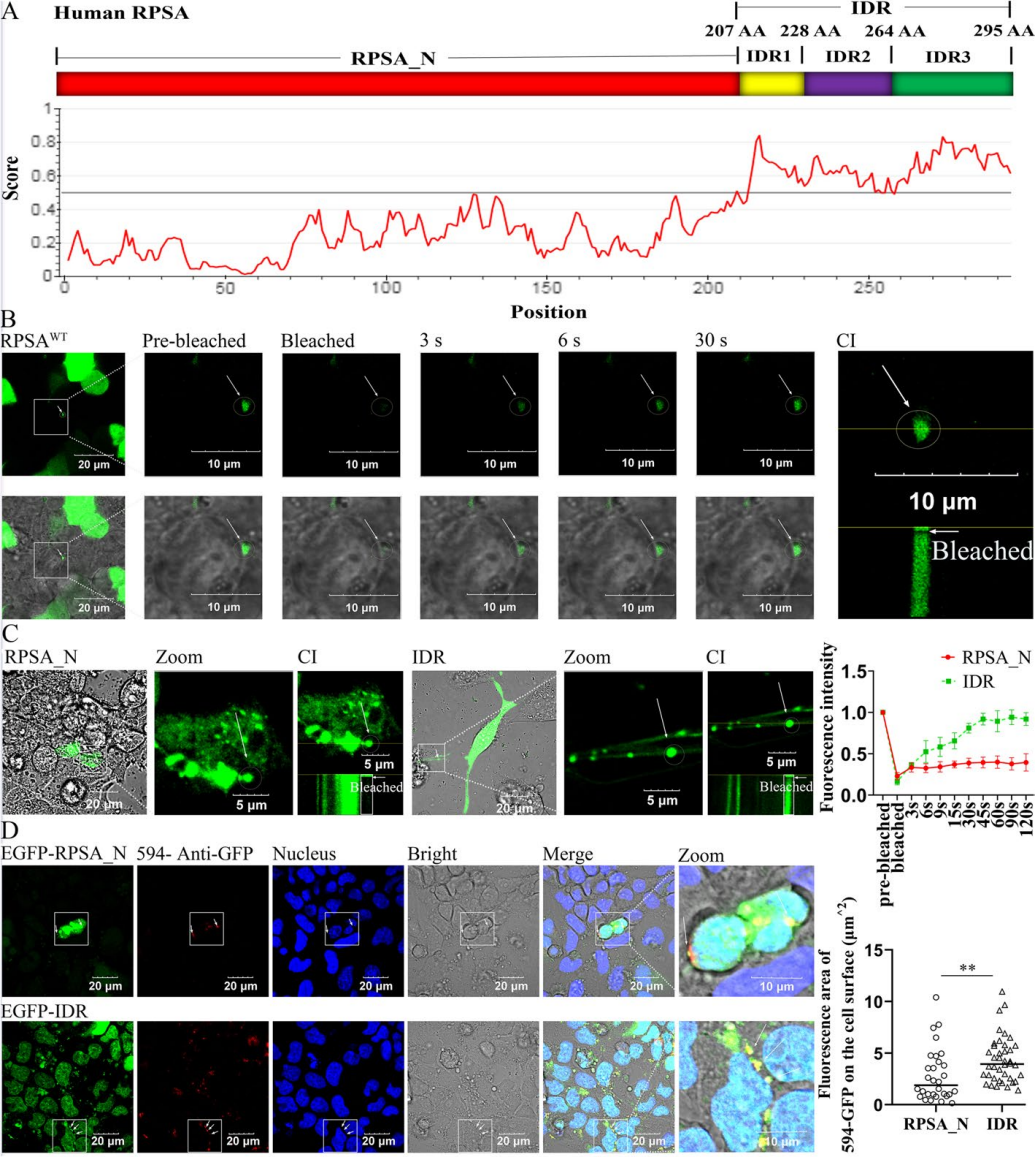
IDR of RPSA is necessary for liquid-like condensation in HEK-293 T cells. **A** The C-terminal (207 AA-295 AA) of human RPSA is intrinsically unstructured (https://iupred2a.elte.hu/). **B**-**D** HEK-293 T cells overexpressing human EGFP-RPSA^WT^, EGFP-RPSA_N_^1 AA−206 AA^ and EGFP-IDR^207 AA−295 AA^ for 24 h. **B** FRAP analyses of RPSA condensates. A typical condensate is shown (scale bar = 20 μm) and magnified (scale bar = 10 μm). **C** FRAP analyses of RPSA_N_ and IDR condensates. FRAP images of condensates are shown (scale bar = 20 μm) and magnified (scale bar = 5 μm). Quantification of FRAP on condensates (*n* = 5 condensates) is shown (mean ± SD). (B and C) "CI" denotes a continuous imaging combination. "Bleached" denotes the extent of fluorescence quenching, and subsequent continuous shooting is used to measure the extent of fluorescence recovery. **D** Representative confocal images are shown (scale bar = 20 μm) and magnified (scale bar = 10 μm). The cell membrane was not permeabilized while labeling GFP proteins with 594-conjugated antibodies. Areas of 594-GFP on the cell surface were used for quantitative analysis by Image J (RPSA_N_ group: *n* = 30 cells, IDR group: *n* = 40 cells, mean ± median). ** for *P* < 0.01; Mann–Whitney test

Our recent research found that SS2 promoted the translocation of RPSA from the intracellular compartment to the membrane and aggregation [3]. In order to screen the domains that drive the transfer of RPSA from the cytosol to the cell surface, we used the IUPred2A to analyze the RPSA amino acid (AA) sequence and found that the C-terminus (RPSA_C) has an intrinsically disordered region (IDR^207 AA−295 AA^), which contains two segments with potential high capability for LLPS including IDR1^207 AA−228 AA^ and IDR3^264 AA−295 AA^ (Fig. 1A). To explore the LLPS capability of RPSA, we found that it could form spherical puncta or granules in human cerebral microvascular endothelial cell line (HCMEC/D3) and in vitro (Additional file 1: Fig. S1A and B). Fluorescence recovery after photobleaching (FRAP) experiments further found that the signal of EGFP-RPSA in condensates or droplets (white arrows) can recover significantly after photobleaching (Fig. 1B), suggesting the RPSA condensates exhibit liquid-like properties with dynamic internal rearrangement and internal–external exchange of molecules.

To further examine the role of IDR in RPSA condensation, we prepared plasmids expressing EGFP-RPSA_N_^1 AA−206 AA^ and EGFP-IDR^207 AA−295 AA^ which were transfected to human embryonic kidney 293 T (HEK-293 T) cells. The recovery capabilities of condensates were examined after photobleaching by FRAP experiments. EGFP-RPSA_N_^1 AA−206 AA^ condensates were mostly not recovered, but EGFP-IDR^207 AA−295 AA^ had the capacity to recover after photobleaching (Fig. 1C, white arrows indicate RPSA condensates). The results suggest that the RPSA IDR^207 AA−295 AA^ likely plays an important role in regulating liquid-like condensation (Fig. 1C right), whereas RPSA_N_ condensates within cells lack liquid fluidity (later referred to as solid aggregates, solid-like aggregation) (Fig. 1C left). In addition, immunofluorescence (IF) images showed that EGFP-IDR^207 AA−295 AA^ formed distinct condensates on the cell surface (Fig. 1D bottom), whereas EGFP-RPSA_N_^1 AA−206 AA^ was highly enriched inside the cells (Fig. 1D top). This indicates that IDR favors RPSA translocation from the cytosol to the cell surface.

### IDR1 (LCD) plays a key role in attenuating intracellular RPSA solid-like condensation

**Fig. 2:**
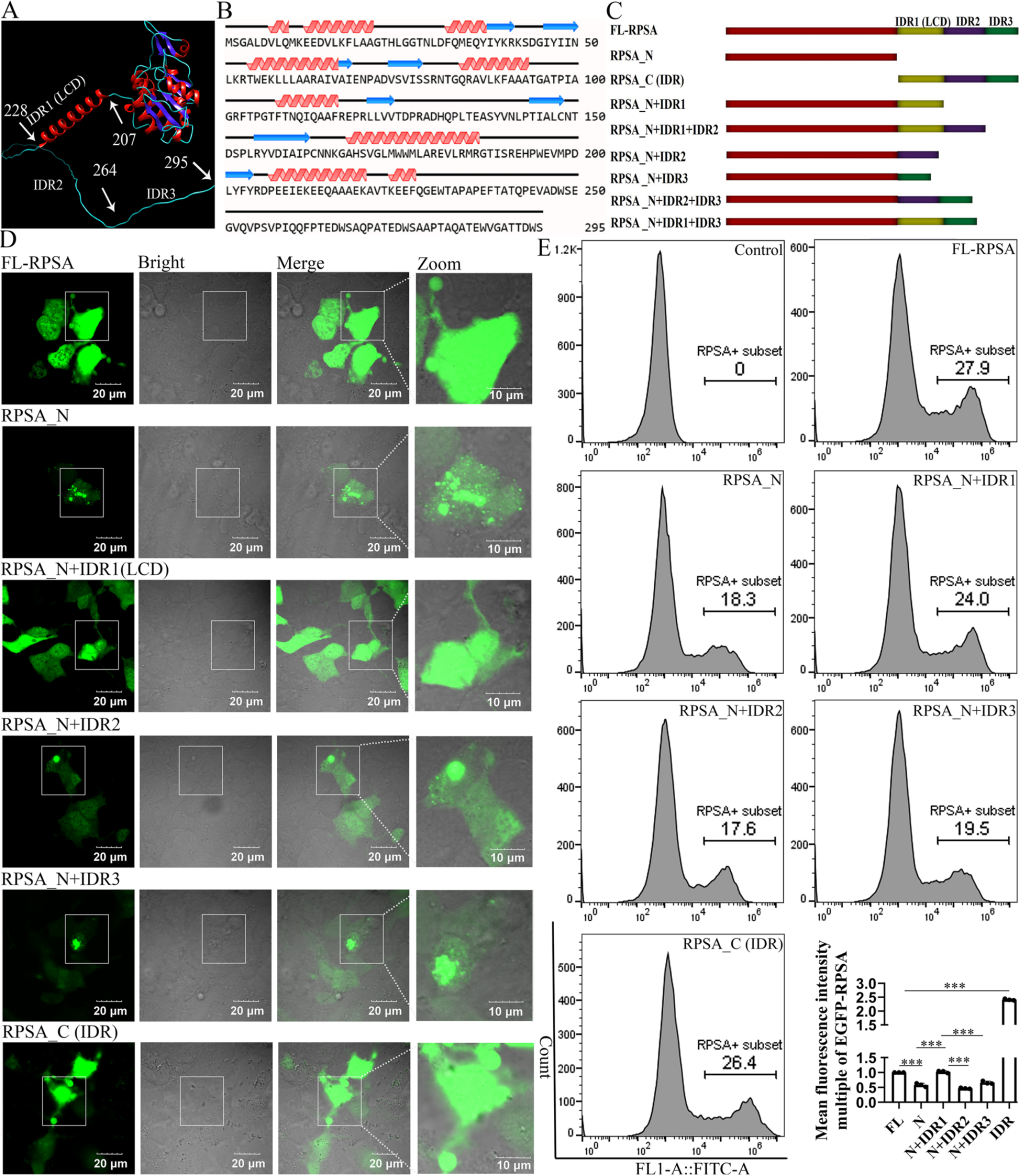
Liquefaction and protein amount of intracellular RPSA_N_ are improved by LCD located in the IDR. **A** The tertiary structure of the human RPSA was analyzed by using the Alphafold Protein Structure Database (https://alphafold.ebi.ac.uk/). **B** The secondary structure of the human RPSA was analyzed using online software (https://www.novopro.cn/). **C** Schematic diagram of RPSA and truncated protein constructs. **D** and** E** HEK-293 T cells overexpressing EGFP-RPSA^WT^ and truncated derivatives for 24 h. **D** Representative confocal images are shown (scale bar = 20 μm) and magnified (scale bar = 10 μm). **E** The mean fluorescence intensity was analyzed by flow cytometry. Quantification was performed by FlowJo as mean ± SD (*n* = 3 independent experiments). *** for *P* < 0.001; one-way ANOVA with Tukey’s test

IDRs of proteins have been implicated in condensate formation in cells, but whether or not an IDR is involved in phase separation still depends on the physical chemistry encoded within its amino acid sequence. Based on RPSA 3D-structure prediction and primary sequence analysis, it was found that the IDR1^207 AA−228 AA^ sequence was a α helical structure of a low complexity domain (LCD), while IDR2^229 AA−263 AA^ and IDR3^264 AA−295 AA^ were unstructured (Fig. 2A and B). Consequently, we constructed related RPSA truncated and/or built-up proteins in HEK-293 T cells transfected with corresponding plasmids (Fig. 2C). It was discovered that the RPSA_N_^1 AA−206 AA^ protein solidified to form solid aggregates inside cells, and that the only way to decrease this intracellular solid-like aggregation was by connecting it to IDR1^207 AA−228 AA^ (LCD) (Fig. 2D). The effect of IDR1 on RPSA protein production was also verified by comparing the mean fluorescence intensity (MnIx) of various truncated bodies. We found that the MnIx of the positive cell population for EGFP-RPSA_N_^1 AA−206 AA^ was considerably lower than that of the positive cell population for EGFP-RPSA^WT^, while the EGFP-RPSA_N+IDR1_^1 AA−228 AA^ positive cell population exhibited a MnIx that was significantly higher than the EGFP-RPSA_N_^1 AA−206 AA^ positive cell population (Fig. 2E). These findings demonstrate that IDR1 (LCD) is a crucial domain for enhancing the degree of RPSA protein production and can facilitate the liquefaction of RPSA_N_ inside cells.

### EE residues in IDR1 (LCD) are crucial for promoting RPSA interior fluidity and protein production

**Fig. 3:**
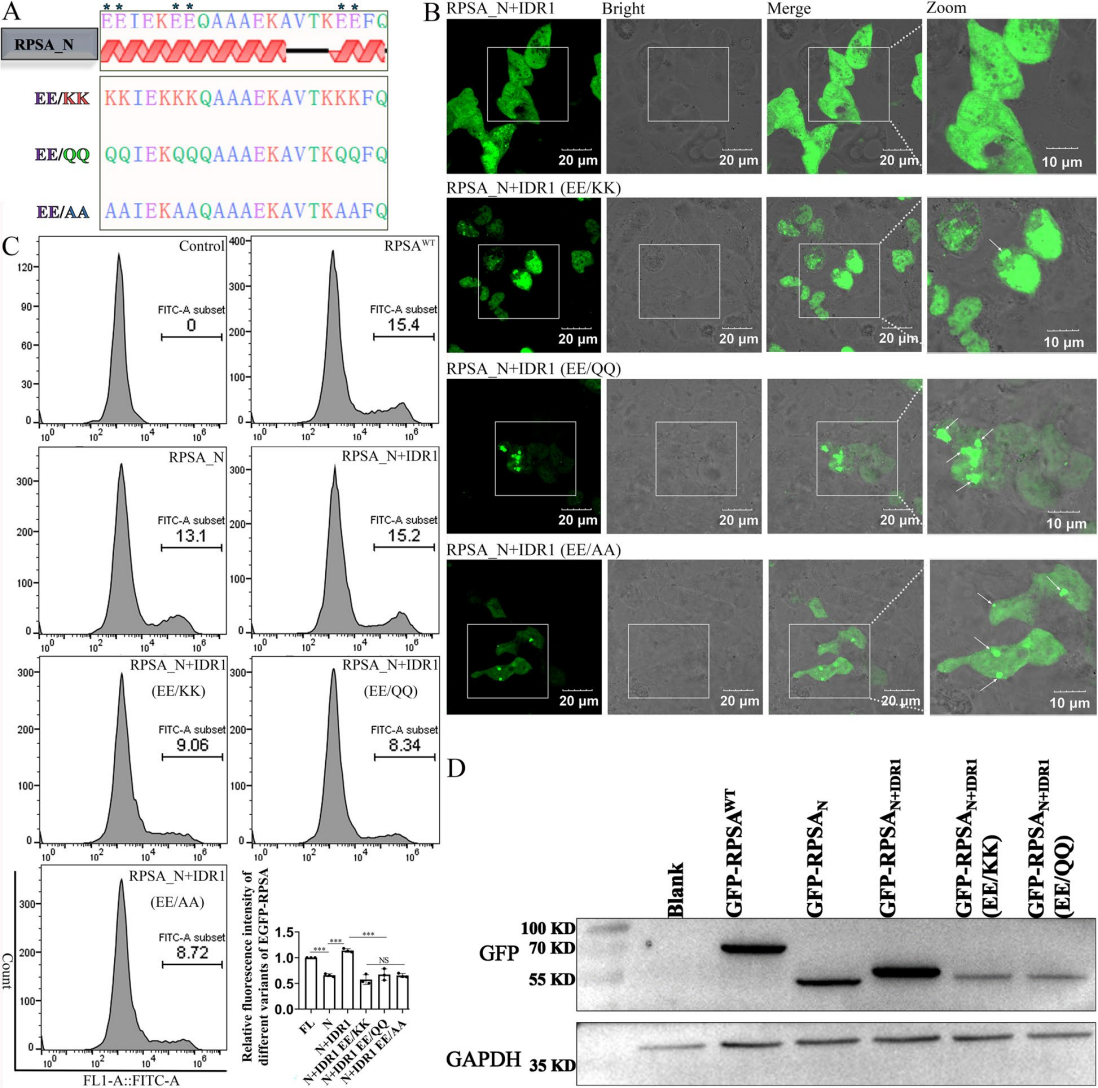
Negatively-charged residues located in the LCD are necessary for enhancing RPSA_N_ liquefaction and protein production. **A** Schematic diagram of human RPSA_N+IDR1_ and mutant derivatives. **B-D** HEK-293 T cells overexpressing EGFP-RPSA^WT^ and its mutant derivatives for 24 h. **B** The intracellular proteins were analyzed by immunofluorescence. Representative confocal images are shown (scale bar = 20 μm) and magnified (scale bar = 10 μm). **C** The mean fluorescence intensity was analyzed by flow cytometry. Quantification was performed by FlowJo as mean ± SD (*n* = 3 independent experiments). NS for not significant, *** for *P* < 0.001; one-way ANOVA with Tukey’s test. **D** The protein production level was detected by western blotting using the antibody against GFP

The RPSA IDR is characterized by a sequence of polar negatively-charged residues enriched in aspartic acid (Asp, D) and glutamic acid (Glu, E). In particular, the LCD contains three repeated units of negatively charged residues (EE repeats). In order to further determine whether EE repeats contribute to LCD functionality, we constructed three mutants based on the schematic design in HEK-293 T cells through plasmid transfection (Fig. 3A). To alter the charge and hydrophilic characteristics of the IDR, we substituted these negatively-charged residues with polar positively-charged residues (Lys, K), polar-uncharged residues (Gln, Q), and hydrophobic residues (Ala, A) (Fig. 3A). Immunofluorescent analyses found that EGFP-RPSA_N+IDR1 (EE/QQ)_ or EGFP-RPSA_N+IDR1 (EE/AA)_ mutants had a larger degree of aggregation than EGFP-RPSA_N+IDR1_ and EGFP-RPSA_N+IDR1 (EE/KK)_ (Fig. 3B, white arrows). The results suggest that the liquefaction of RPSA_N_ is aided mainly by the EEs or KKs residues in LCD. Since the LCD is a crucial region that promotes higher levels of RPSA protein production (Fig. 2E), flow cytometry was also used to measure the MnIx of EGFP-RPSA_N+IDR1_^1 AA−228 AA^ and associated mutations. In comparison to EGFP-RPSA_N+IDR1_^1 AA−228 AA^, it was found that the MnIx of each mutant was much reduced (Fig. 3C). Additionally, the western blotting (WB) results also found that the protein production levels of EGFP-RPSA_N+IDR1 (EE/KK)_ and EGFP-RPSA_N+IDR1 (EE/QQ)_ were much lower than those of EGFP-RPSA_N+IDR1_^1 AA−228 AA^ (Fig. 3D). The results suggest that the production of the RPSA protein as is its liquefaction.

### ENO promotes RPSA liquid-like condensation

**Fig. 4:**
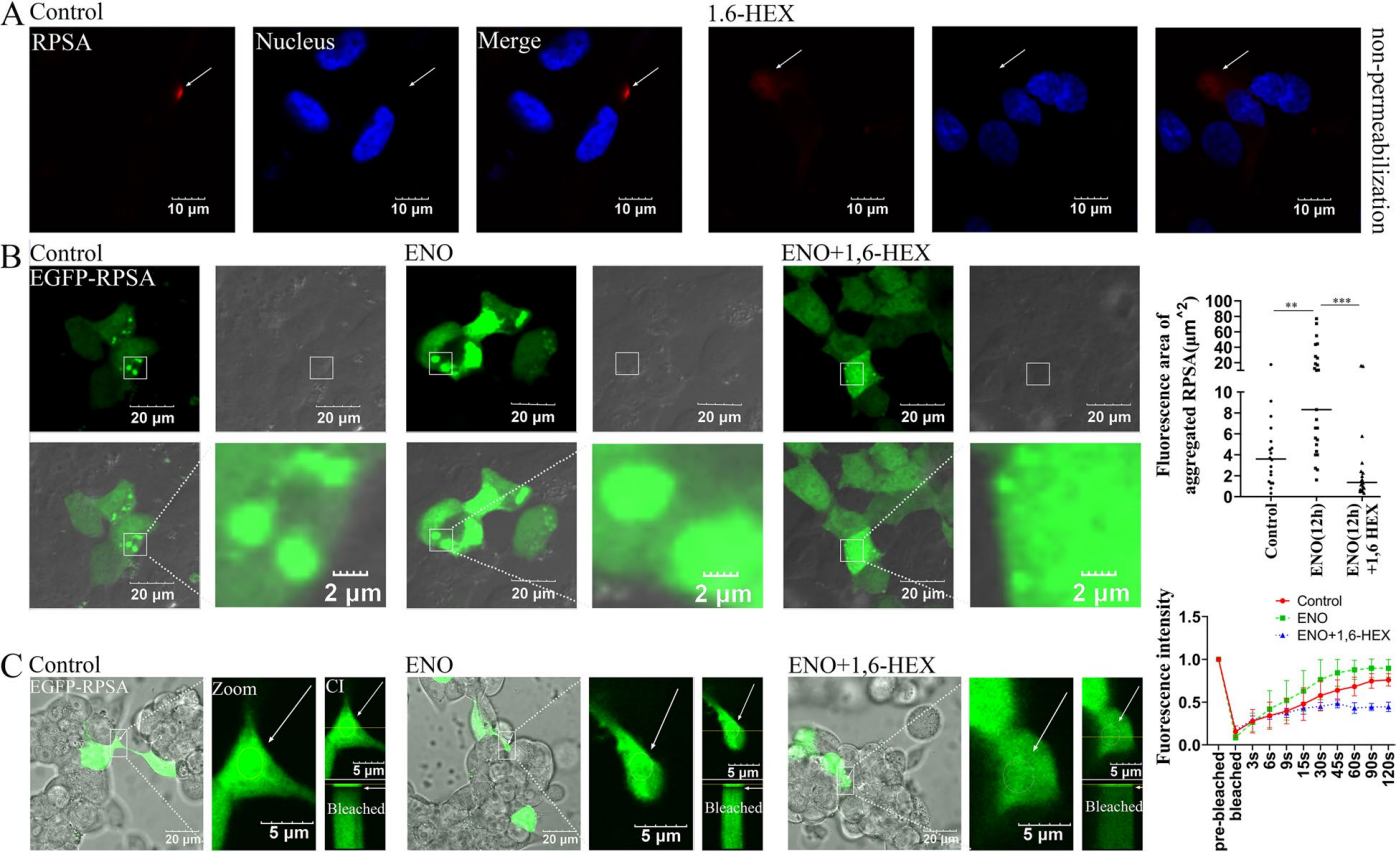
ENO stimulation promotes RPSA liquid-like condensation. **A** HCMEC/D3 cells were treated with or without 1,6-Hex. Immunofluorescence shows the degree of aggregation of RPSA on the cell surface (white arrows). Representative confocal images are shown (scale bar = 10 μm). **B **and** C** HEK-293 T cells overexpressing EGFP-RPSA.^WT^ for 12 h, and then ENO (20 μg/mL) protein was added to the transfected cells. After 12 h, cells were treated with or without 1,6-Hex. **B** Immunofluorescence analysis of the size of RPSA condensates (white arrows show condensates). Representative confocal images are shown (scale bar = 20 μm) and magnified (scale bar = 2 μm). The area of condensates was used for quantitative analysis by Image J (Control group: *n* = 18 condensates, ENO group: *n* = 25 condensates, ENO + 1,6-Hex group: *n* = 18 condensates, mean ± median). ** for *P* < 0.01, *** for *P* < 0.001; one-way ANOVA with Kruskal–Wallis test. **C** FRAP analyzes indicated the recovery ability of condensates. FRAP images are shown (scale bar = 20 μm) and magnified (scale bar = 5 μm). In Fig. 1, the symbols for "CI" and "Bleached" were depicted. Quantification of FRAP on condensates (*n* = 10 condensates) is shown (mean ± SD)

1,6-Hexanediol (1,6-Hex) is a widely used tool to probe LLPS in cells, and acts by disruption of phase separation assemblies. RPSA condensates in HCMEC/D3 cells were considerably diffused following treatment with 1,6-Hex (Fig. 4A, white arrows). Previously, we determined that SS2-ENO promotes increased RPSA protein expression [3]. Here, the impact of ENO on the production of RPSA condensates was also investigated. 1,6-Hex treatment greatly decreased the size of intracellular EGFP-RPSA condensates in HEK-293 T cells whereas ENO stimulation alone increased it significantly (Fig. 4B). The fluidity of the RPSA condensate on the cell surface was also analyzed. In contrast to 1,6-Hex, it was found that ENO considerably improved the capacity of EGFP-RPSA condensates to recover after photobleaching (Fig. 4C, white arrows). Thus, ENO increases RPSA protein expression and facilitates RPSA/ENO liquid-like condensation that is sensitive to 1,6-Hex.

### LCD is a prerequisite for ENO and RPSA to form co-condensates but also require IDR2 and/or IDR3

**Fig. 5:**
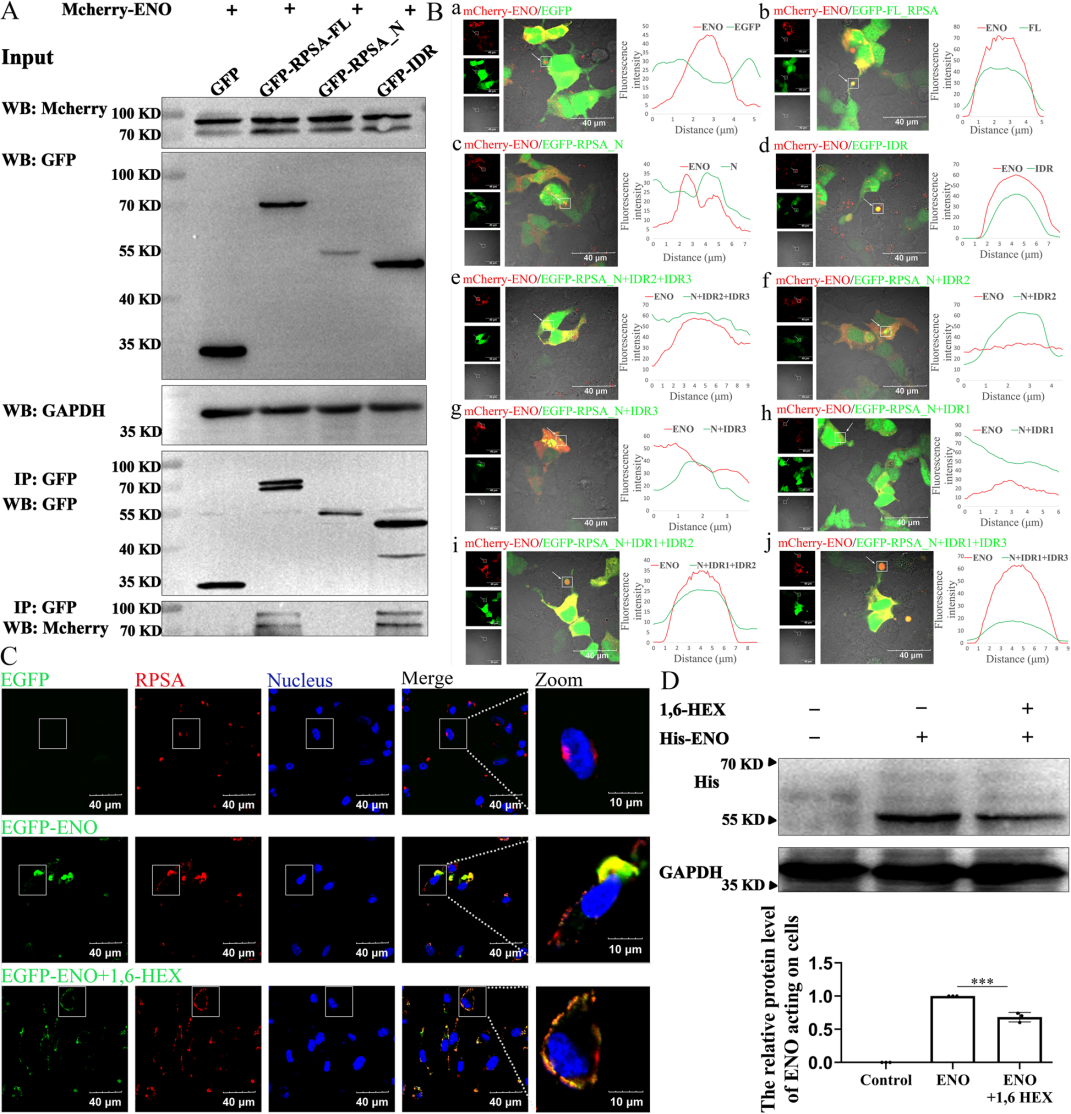
ENO binds to the RPSA IDR to form co-condensates. **A** and** B** HEK-293 T cells co-overexpressing mCherry-ENO and the indicated proteins for 36 h. **A** Cell lysates were immunoprecipitated with the antibody against GFP, followed by western blot analysis using an antibody against mCherry. **B** Co-localization of ENO and different RPSA truncations were observed by immunofluorescence. Representative images were shown (scale bar = 40 μm, left). Co-localization analysis of the selected arrowed areas used Image J (ENO and indicated protein co-localization analysis, right). **C** and **D** EGFP-ENO or ENO (20 μg/mL) protein was added to HCMEC/D3 cells. After 24 h, cells were treated with or without 1,6-Hex. **C** Adhesion of ENO to the cell surface was determined by immunofluorescence staining using the antibody against RPSA. Representative confocal images are shown (scale bar = 40 μm) and magnified (scale bar = 10 μm). **D** The amount of ENO protein acting on the cell was determined by western blotting using the antibody against his. Quantification was performed by Image J as mean ± SD (*n* = 3 biologically independent samples). ** for *P* < 0.01; one-way ANOVA with Tukey’s test

IDR is the main extracellular region of RPSA [8], ENO and IDR of RPSA interact, as shown by Co-immunoprecipitation (CO-IP) (Fig. 5A). Co-localization of ENO and individual RPSA truncations (see Fig. 2C for details) further confirmed ENO-RPSA co-condensation. In contrast to the EGFP protein, mCherry-ENO and EGFP-RPSA were clearly co-localized as spherical condensates (Fig. 5B a and b), but mCherry-ENO and intracellular EGFP-RPSA_N_ solid aggregates seldom co-localized (Fig. 5B c). The co-localization of mCherry-ENO and EGFP-IDR condensates was clear (Fig. 5B d). We also discovered that, in the absence of the IDR1, EGFP-RPSA_N+IDR2+IDR3_ (Fig. 5B e), EGFP-RPSA_N+IDR2_ (Fig. 5B f) and EGFP-RPSA_N+IDR3_ (Fig. 5B g) were associated mainly with solid-state aggregation inside the cells. mCherry-ENO was weakly co-localized with these intracellular solid aggregates (Fig. 5B e–g). ENO and RPSA_N+IDR1_ were not colocalized (Fig. 5B h), and IDR1 only weakened the RPSA_N_ solid-like aggregation; no liquid condensate formation was found. However, in the presence of IDR1, spherical liquid condensates were successfully formed with the help of IDR2 and/or IDR3, and RPSA_N was co-localized with mCherry-ENO (Fig. 5B i and j). Thus, ENO and RPSA form liquid condensates and these are facilitated by IDR1 + IDR2 and/or IDR3.

To further verify that ENO binds cells by forming a co-condensate with RPSA, the adhesion of ENO to cells with or without 1,6-Hex treatment was determined. ENO adhered to the cell surface in clumps when 1,6-Hex was not present, but was scattered around cells when 1,6-Hex was added (Fig. 5C). The 1,6-Hex treatment greatly decreased the quantity of ENO protein attached to cells, as further evidenced by western blotting (Fig. 5D). This suggests that ENO binding to cells is favored by RPSA condensates. In brief, ENO stimulation encourages RPSA protein production, which in turn facilitates RPSA liquid-like condensation and translocation, resulting in the formation of ENO-RPSA co-condensates on cell membranes.

### ENO promotes interaction of RPSA with vimentin (VIM) on the cell membrane

**Fig. 6:**
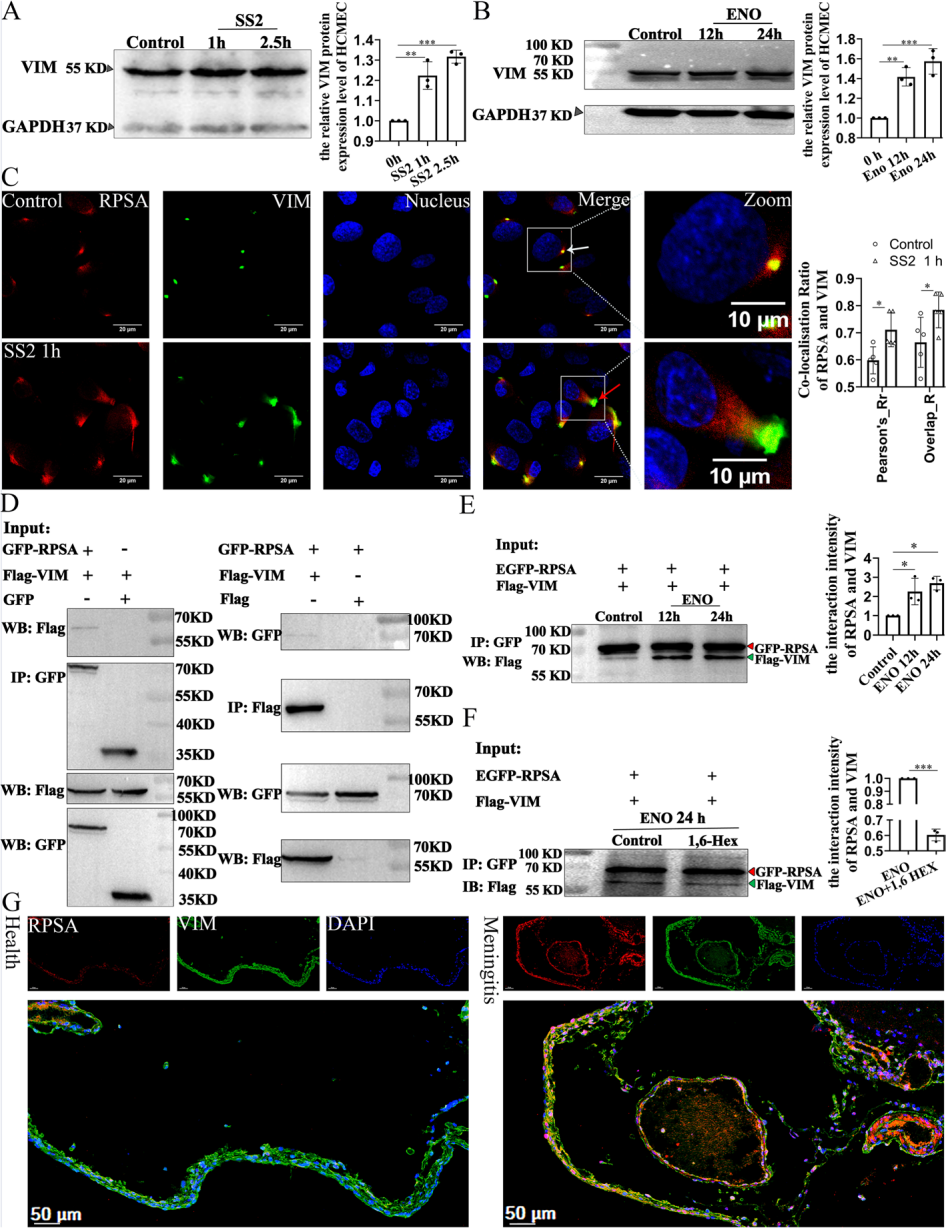
ENO-RPSA-VIM forms co-condensates on the cell membrane. **A** and** B** After the corresponding time of SS2 or ENO (20 μg/mL) protein stimulation for HCMEC/D3 cells, cell lysates were used for western blot analysis. Quantification was performed by Image J (*n* = 3 biologically independent samples, mean ± SD). **C** After SS2 infection of HCMEC/D3 cells for 1 h, the localization of RPSA and VIM on the cell surface was observed by immunofluorescence staining using the antibodies against RPSA and VIM. Representative confocal images are shown (scale bar = 20 μm) and magnified (scale bar = 10 μm). Pearson and Overlap Coefficients were quantitatively analyzed by Image J (*n* = 5 fields in each group, a field contains about 8 cells, mean ± SD). **D—F** HEK-293 T cells were co-transfected with the indicated plasmids co-expressing GFP-RPSA and FLAG-VIM proteins (GFP-RPSA and FLAG, GFP and FLAG-VIM). **D** After 36 h, cell lysates were immunoprecipitated with the antibody against GFP or FLAG, followed by western blot analysis using the indicated antibodies against FLAG or GFP. **E** and **F** After 12 h, ENO (20 μg/mL) protein was added to the transfected cells for stimulation. After stimulation for the indicated times, cells were treated with (**E**) or without (**F**) 1,6-Hex. Cell lysates were immunoprecipitated with antibody against GFP, followed by western blot analysis using the antibody against FLAG. Quantification was performed by Image J as mean ± SD (*n* = 3 biologically independent samples). **G** Multiples fluorescence immunohistochemistry analyses of the co-localization of RPSA and VIM proteins from brain tissue of healthy and SS2-infected piglets. Representative stained tissue slices were shown (scale bar = 50 μm). **A-C, E** and **F** * for *P* < 0.05, ** for *P* < 0.01, *** for *P* < 0.001; (A, B and E) one-way ANOVA with Tukey’s test, (C and F) two-tailed unpaired Student’s t-test

Through pull-down and mass spectrometry sequencing, 47 proteins were found that potentially interact with RPSA in ENO-RPSA co-condensates (Additional file 1: Fig. S2A and B). We found that intermediate filament cytoskeleton organization was strongly correlated with these proteins (Additional file 1: Fig. S2C). For verification of the relationship between RPSA and Vimentin (VIM), the protein expression level of VIM after SS2 infection or ENO stimulation was analyzed. Both SS2 infection and ENO stimulation also markedly increased the protein expression level of VIM (Fig. 6A and B). Under normal physiological conditions, RPSA and VIM were co-localized as a dot-like aggregation on the cell membranes (Fig. 6C, white arrow). After SS2 infection, co-localization of RPSA and VIM dramatically increased, particularly where bulges were observed; these were in contrast to uninfected cells (Fig. 6C, red arrow). Furthermore, we also analyzed the relationship between RPSA and VIM in response to ENO stimulation of HEK-293 T cells which were transfected with the indicated plasmids. The results showed that ENO significantly promoted the interaction strength of RPSA and VIM (Fig. 6D and E), but 1,6-Hex treatment greatly reduced the strength of their interaction (Fig. 6F). Staining of piglet brain tissue from healthy and experimental SS2 meningitis was performed using multicolor fluorescence immunohistochemistry experiments (mIHC). After SS2 infection, it was discovered that RPSA and VIM protein expression levels were increased (Additional file 1: Fig. S3), and they were clearly co-localized in tissue (Fig. 6G). These results suggest that SS2 infection induces the ENO-stimulated incorporation and thus concentration of RPSA-VIM complex on the cell surface.

### ENO-RPSA-VIM complexes elevate intracellular calcium ion levels

**Fig. 7:**
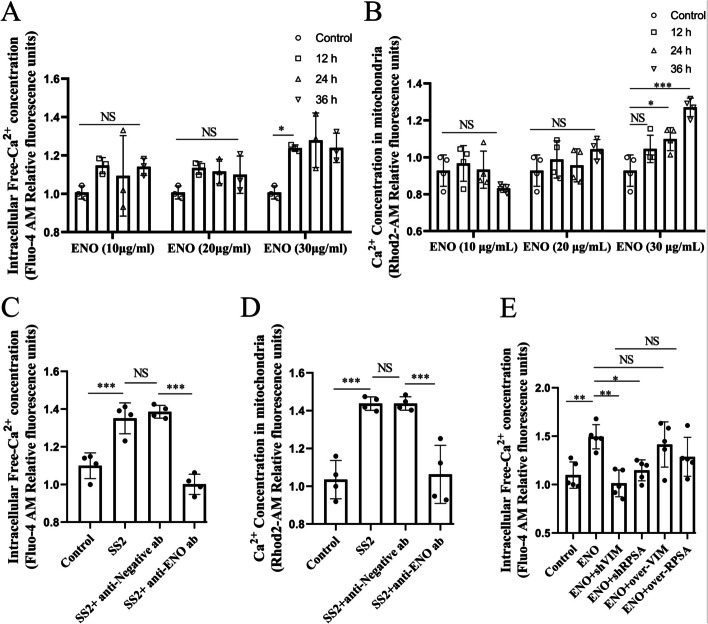
ENO stimulation elevated the content of intracellular Ca^2+^ mediated by RPSA-VIM co-condensates. **A** and** B** HCMEC/D3 cells were stimulated for the indicated times using the indicated final concentration of ENO protein. The intracellular Ca^2+^ content (**A**) or mitochondrial Ca^2+^ content **(B)** was detected. **C** and **D** The indicated serum and SS2 were mixed and added together to the HCMEC/D3 cells. After 2 h, intracellular Ca^2+^ content **(C)** or mitochondrial Ca^2+^ content **(D)** was then detected. **E** After HEK**-**293 T cells overexpressing or inhabiting the indicated protein expression level for 12 h, ENO (30 μg/mL) protein was added to the transfected cells. After 24 h, the intracellular Ca^2+^ content was then detected. Data represent the mean ± SD (**A**, *n* = 3 biologically independent samples. **B-D**, *n* = 4 biologically independent samples. **E**, *n* = 5 biologically independent samples). NS for not significant, * for *P* < 0.05, ** for *P* < 0.01, *** for *P* < 0.001; one-way ANOVA with Tukey’s test

VIM exerts an important protective effect on mitochondria [11], and VIM filaments also connect adjacent cells together (Additional file 1: Fig. S4A, red arrows). However, notably 2.5 h after infection, SS2 substantially damaged VIM filaments among the cells, and morphologically swollen mitochondria were observed (Additional file 1: Fig. S4A, white arrows). ENO stimulation appears to lower mitochondrial activity (Additional file 1: Fig. S4B). Mitochondrial and intracellular Ca^2+^ concentrations were determined and it was found that ENO stimulation elevated Ca^2+^ content (Fig. 7A and B). When the activity of ENO was blocked, the effect of SS2 on Ca^2+^ concentration was significantly inhibited (Fig. 7C and D). Additionally, the effect of SS2 on mitochondrial membrane potential (MMP) and reactive oxygen species (ROS) level was also significantly suppressed (Additional file 1: Fig. S4C and D). However, when RPSA and VIM protein expression were interfered with using shRNA, Ca^2+^ levels were considerably suppressed after ENO stimulation (Fig. 7E). These results indicate that ENO-RPSA-VIM likely forms co-condensates that cooperatively damage mitochondria while increasing intracellular Ca^2+^ levels.

### *Ca*^*2*+^*facilitates the RPSA condensate formation and aggravates the cytotoxic activity against host cells*

**Fig. 8:**
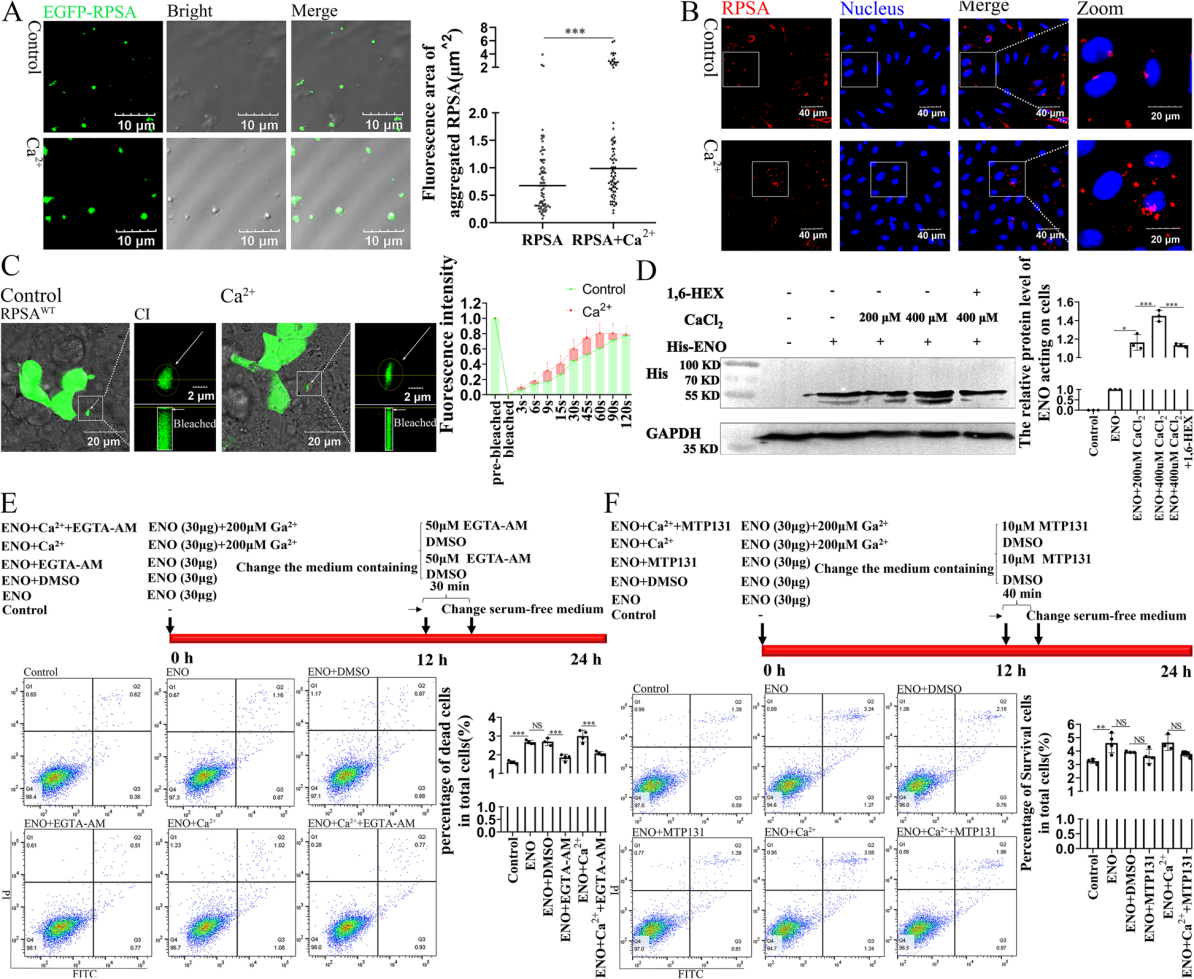
Ca^2+^ improve the capacity of ENO to cause apoptosis. **A** Representative images of droplet formation of GFP-RPSA in vitro (Scale bar = 10 μm). One group additionally had added 3 mM Ca^2+^. The area of RPSA condensates were used for quantitative analysis by Image J (RPSA group: *n* = 72 condensates, RPSA + Ca^2+^ group: *n* = 83 condensates, mean ± median). Mann–Whitney test. **B** After HCMCE/D3 cells were pretreated with or without Ca^2+^ (200 μM) medium for 24 h, RPSA on the cell surface was observed by immunofluorescence staining using the antibody against RPSA. Representative confocal images are shown (scale bar = 40 μm) and magnified (scale bar = 20 μm). **C** HEK-293 T cells overexpressing EGFP-RPSA^WT^ for 12 h. The transfected cells were pretreated as described in (**B)**. FRAP analyses of condensates in each group. Representative FRAP images are shown (scale bar = 20 μm). In Fig. 1, the symbols for "CI" and "Bleached" were depicted. Quantification of FRAP as mean ± SD (*n* = 10 condensates). **D** After HCMCE/D3 cells were pretreated as described in (**B**), ENO (30 μg/mL) protein was added to the cells. After 24 h, cells were treated with or without 1,6-Hex. The amount of ENO protein acting on the cell was quantified by western blot analysis. Quantification was performed by Image J as mean ± SD (*n* = 3 biologically independent samples). **E **and **F** The procedure followed by Ca^2+^ chelating agent experiments (**E a**) and mitochondrial protective agent experiments (**F a**) is shown. (**E b, F b**) Flow cytometry analyzed that the death level of cells. The ratio of dead cells to total cells in the indicated conditions was analyzed by FlowJo as mean ± SD (*n* = 4 biologically independent samples). **A**,** D-F** NS for not significant, * for *P* < 0.05, *** for *P* < 0.001; **D-F** one-way ANOVA with Tukey’s test

In vitro testing of the impact of Ca^2+^ on RPSA condensate production revealed that it may greatly enhance the size of the condensates (Fig. 8A). The amount of RPSA condensates on the cell membranes of HCMCE/D3 cells greatly increased with the addition of more Ca^2+^ (Fig. 8B). Additionally, FRAP analysis found that Ca^2+^ improved the capacity of EGFP-RPSA condensates to recover after photobleaching (Fig. 8C). Western blotting analysis showed that addition of Ca^2+^ also increased the amount of ENO protein acting on cells, which was still sensitive to the 1,6-Hex treatment (Fig. 8D). Our previous studies have shown that the capacity of ENO to bind to RPSA results in cell apoptosis [5], so we sought to investigate whether the addition of Ca^2+^ can amplify the effect of ENO on cell apoptosis. ENO stimulation for 24 h primarily caused apoptosis (Additional file 1: Fig. S5A). A final concentration of 200 μM Ca^2+^ did not trigger apoptosis, but did when mixed with ENO (Additional file 1: Fig. S5B). Furthermore, we employed ENO to stimulate cells treated with an intracellular Ca^2+^ chelating agent and subsequently analyzed changes in apoptosis levels by flow cytometry. We found that lessening the intracellular Ca^2+^ level using a chelator during ENO stimulation significantly lowered the rate of cell death (Fig. 8E), validating the relation between increased intracellular Ca^2+^ and apoptosis under ENO stimulation. Additionally, the application of mitochondrial protectors during ENO stimulation reduced cell death to some extent (Fig. 8F). These findings suggest that intracellular Ca^2+^ facilitates ENO-RPSA-VIM co-condensate formation and stimulates apoptosis.

## Discussion

**Fig. 9:**
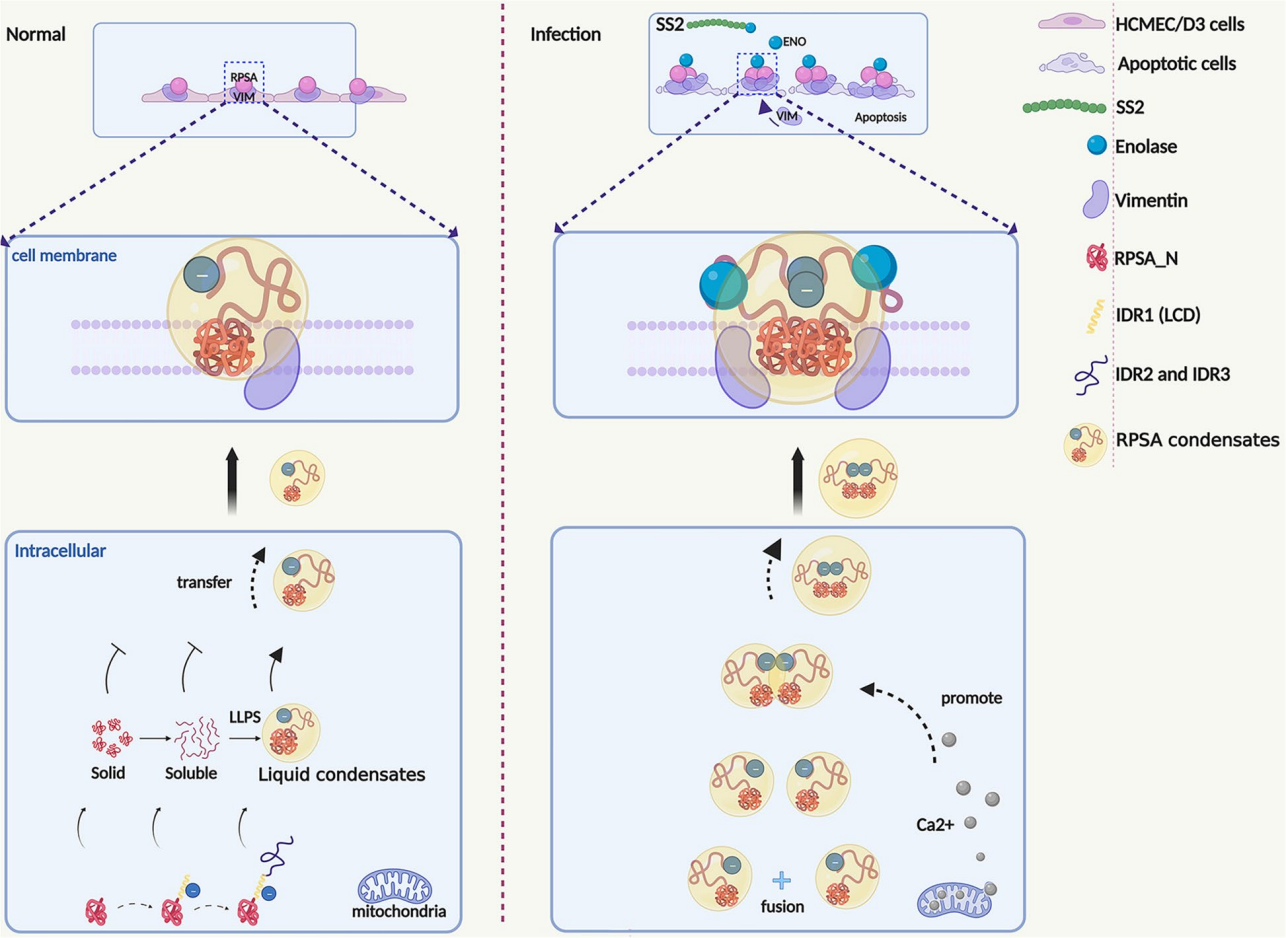
Model figure. RPSA translocation from the intracellular environment (cytosol) to the cell surface in the form of liquid-like condensates driven by its IDR. ENO binds to the IDR of RPSA and promotes its interaction with RPSA and VIM on cell membrane. The incorporation and concentration of ENO-RPSA-VIM complexes increase the cellular Ca^2+^ level, and consequently promotes the RPSA condensate formation and induces HBMEC apoptosis. Images were created with Biorender.com

Due to its location as receptors on the cell membrane surface, RPSA plays a crucial part in mediating pathogenic microbial infection. This study has shown that RPSA localization and condensation on the cell surface depends on negatively-charged residues in IDR1 (LCD). The SS2 virulence factor ENO acts on IDR of RPSA to promote the formation of biomolecular condensates containing protein molecules such as RPSA and VIM, which further induces cell apoptosis. Even, the increased intracellular Ca^2+^ caused by ENO is conducive to the RPSA condensate formation and cell apoptosis (Fig. 9, Images were created with Biorender.com).

### LCD which is located in the IDR is beneficial to the RPSA translocation

RPSA is mainly composed of two parts, the RPS2-like globular domain at the N-terminus and the IDR at the C-terminus [7]. The propensity for protein aggregation is a ubiquitous and seemingly inevitable property of the proteome. This intrinsic tendency to aggregate is directly related to globular stability [12]. The RPS2-like domains of RPSA were globularly aggregated, and this hydrophobic aggregation also explained why the aggregated RPS2 domains of RPSA could not be recovered after photobleaching in our study (Fig. 1). Intrinsically disordered protein (IDP) domains lacking stable 3D-structures are the only class of naturally occurring polypeptides with significantly reduced predicted aggregation regions during long-term evolution [12]. The IDR of RPSA can reduce the RPSA_N_ solid-like aggregation and favors the translocation to the cell surface (Fig. 1), which may add additional support to the theory. LCD are additionally known as prion-like domains, because they have been associated with an ability to spread among yeast populations in a prion-like manner [13]. Martin et al. show that the uniform distribution of aromatic residues along LCDs favors solubility and LLPS over aggregation [14]. In our study, glutamic acid (Glu, E) residues in LCD are crucial for promoting RPSA interior fluidity, rather than allowing RPSA to aggregate within cells (Figs. 2 and 3). This shows that LCD has the property of favoring protein spread and solubility. The budding yeast translation termination factor Sup35 is also a yeast prion protein. LLPS of Sup35 was mediated by the N-terminal prion domain and regulated by the M domain. In the absence of a disordered NM domain, the C domain also undergoes irreversible aggregation. Interestingly, this irreversible aggregation can be reduced when the M domain exists [15]. Both RPSA and Sup35 have domains that weaken their own hydrophobic aggregation, and the properties may give them a wide range of functions. It turns out that the C-terminus of RPSA is a key extracellular region for interaction with 18S rRNA, immunoglobulins, carbohydrates, and laminin 1, among others [8]. The C-terminal 263aa-282aa region is also a receptor for some pathogens [6]. Our research has demonstrated that the IDR of RPSA is also the main region for ENO binding (Fig. 5). We hypothesize that in order to fulfill receptor function, RPSA must be transferred from an intracellular location to the cell surface, and IDR is crucial for this. Min et al. believe that phase separation may be a general mechanism for protein sorting [16]. Our research adds further support to this claim.

### RPSA and VIM jointly mediate the infectious process of pathogenic microorganisms

Vimentin (VIM) is an intermediate filament protein of meninges and endothelial cells. Recently, Jiu et al. reported that VIM is a cage for ZIKV intracellular replication [17]. The intracellular domain (1–85 AA) of RPSA has recently been shown to bind to the membrane-bound E protein of Zika virus (ZIKV) to reduce the ubiquitination of the E protein in a way that attracts the de-ubiquitination enzyme EIF3S5, preventing ZIKV infection [18]. Given that RPSA and VIM are both implicated in the pathogenic process in ZIKV-infected hosts, there may be common pathogenetic pathways. In our study, RPSA_N_ aggregated expression mainly occurred in the cell (Fig. 1). In light of these investigations, we assume that the RPSA N-terminus might play a role in the transmission and activation of downstream intracellular signals during the infectious process of pathogenic microorganisms.

RPSA can engage in interactions with cytoskeletal proteins such as actin, and tubulin [19]. Cytoskeletal proteins themselves have IDR sequences that can form biomolecular condensates [20]. The cytoskeleton can also interact directly with other biomolecular condensates [21]. Although there is no report on the occurrence of LLPS in VIM, it also has an IDR domain [20]. This indicates that VIM possesses an intrinsic factor for phase separation. Our research also confirms that RPSA-VIM forms co-condensates (Fig. 6), but further research is required to determine whether VIM itself can undergo LLPS.

### *Ca*^*2*+^*trigger condensate formation*

There has been shown that RPSA-(225–295) may mask binding sites in RPSA-(2–209), either sterically or through its negative contribution to the electrostatic field [7]. Additionally, it has been found that protein self-interactions caused by electrostatic forces limit the occurrence of LLPS [16, 22]. Therefore, we suggest that N-domain and C-domains of RPSA interact weakly which may limit the occurrence of RPSA LLPS through autoinhibition.

It is well known that VIM proteins have an important role in maintaining the integrity of mitochondrial structure and function [11]. This study confirmed that SS2 infection could destroy VIM filaments among the cells, and morphologically swollen mitochondria were observed in host cells (Additional file 1: Fig. S4). Additionally, SS2 infection significantly altered the levels of MMP, Ca^2+^ concentration, and ROS (Fig. 7 and Additional file 1: Fig. S4). Thus, the application of the MTP-131 during ENO stimulation reduced cell death to some extent (Fig. 8). However, the statistically non-significant reason may be that MTP-131 only ameliorates cellular ROS levels but did not significantly reduce Ca^2+^ content. It is worth noting that the phase separation of proteins can also be regulated by external factors [23]. For example, researchers reported that Ca^2+^ transients on the endoplasmic reticulum (ER) surface trigger LLPS of focal adhesion kinase family interacting protein of 200 kD (FIP200) to autophagosome initiation sites [24]. In this study, we further confirmed that Ca^2+^ can provide an external environmental factor for RPSA condensation during SS2 infection (Fig. 8). The positive-charge carried by Ca^2+^ may have weakened RPSA autoinhibition by electrostatic repulsion, resulting in LLPS enhancement.

Calnexin (CANX) is an ER-associated protein that binds to calcium. It can regulate the Ca^2+^ -handling machinery at the Mitochondria-associated endoplasmic reticulum membrane (MAM) [25]. Our research revealed that RPSA and CANX were co-localized in spherical aggregates [3], which might have an impact on how much Ca^2+^ and RPSA were exposed. Although additional research is necessary, we speculate that increased Ca^2+^ may be connected to the ER. In summary, the increased intracellular Ca^2+^ caused by ENO is conducive to RPSA condensate formation, and the depletion of intracellular Ca^2+^ levels significantly lower the rate of cell death (Fig. 8).

### "Lipid rafts" may has an affinity for RPSA

The formation of "lipid rafts" is considered to occur in two-dimensional space LLPS, which itself has an affinity for IDR-containing proteins. In addition, IDR-containing proteins in "lipid rafts" can also undergo LLPS and form biomolecular condensates [26]. Our previous studies have shown that RPSA and the marker protein of the "lipid raft", caveolin-1 (CAV1), are co-localized on the cell surface under normal physiological conditions [3]. Because RPSA with IDR may be adsorbed by "lipid rafts", this is likely to be the cause of colocalization of RPSA and CAV1 on the cell surface. We have demonstrated that the presence of "lipid rafts" attenuate ENO toxicity [3]. The reason may be that "lipid rafts" adsorb RPSA during SS2 infection, thereby weakening the binding of ENO and RPSA. However, SS2 may make ENO favorable for binding RPSA by destroying "lipid rafts". Further evidence is still required to support this hypothesis.

## Conclusions

We show that ENO interacts with the negatively-charged enriched internally disordered regions (IDRs) of RPSA, driving RPSA transfer from the cytoplasm to the cell surface and promoting RPSA-vimentin (VIM) co-condensation on the membrane. The formation and concentration of ENO-RPSA-VIM complexes increase cellular Ca^2+^ following SS2 infection or ENO stimulation and induce strong cytotoxic activity against HBMECs and, potentially, alter disease progression. This offers a fresh avenue for investigation into the mechanism by which other harmful bacteria that infect hosts via cell surfaces RPSA.

## Methonds

### Plasmid construction and identification

The plasmids used in this study are shown in Additional file 2: Table S1. The corresponding gene fragments generated by PCR amplification used the primers shown in Additional file 2: Table S2. The nucleic acid bases of the *rpsa* gene (NM_002295) were mutated by using chemical synthesis (Sangon Biotech, China). The upstream and downstream fragments were joined together using overlap extension PCR, and primers with restriction sites were used for amplification by Golden Star T6 Super PCR mix (Tsingke Biotech, China). After double-enzyme digestion, the gene fragment of interest was ligated into the corresponding vector. The constructed plasmids were confirmed by PCR amplification and Sanger sequencing (Comate Bioscience, China).

### Proteins expression and purification

The *rpsa* gene was codon-optimized and synthesized according to liu's research by Sangon Biotech (China) [27]. Recombinant ENO, EGFP-ENO and EGFP-RPSA expression, purification and concentration were also determined as described previously [27]. In brief, vectors containing the *eno*, *egfP*-*eno* gene and the optimized *egfP*-*rpsa* genes were both transformed into *Escherichia coli* BL21 (DE3) strain (TIANGEN Biotech, China) for protein expression. A single clone from an overnight Luria broth (LB) agar plate at 37 °C was picked and cultured in 5 ml LB medium containing 50 µg/ml kanamycin. The culture was then transferred to 50 ml LB medium with kanamycin and grown for 2 h at 37 °C. The bacteria were transferred into 500 ml of LB medium in the presence of 50 µg/ml kanamycin and grown to an OD_600_ of 0.8 at 37 °C in a 2 L flask. Expression was induced by adding isopropyl-β-d-1-thiogalactopyranoside (IPTG, Sigma-Aldrich, USA) to a final concentration of 0.5 mM and the cells were continually grown at 37 °C for 6 h. After cell collection, sonication was performed, and the supernatant was used for protein purification. Recombinant proteins were purified from supernatants using Ni–NTA column (Thermo Fisher Scientific, USA) and gradient elution using imidazole. Finally, protein concentration was determined with a BCA protein assay kit (Thermo Fisher Scientific, USA).

### Bacterial strains, cell lines and growth conditions

The virulent SS2 strain JZLQ022 was isolated from the brain tissue of pigs with meningitis [5]. The well-characterized human cerebral microvascular endothelial cell line (HCMEC/D3) was obtained from Professor Yan Chen (College of Life Sciences, Jilin University). Human embryonic kidney 293 T (HEK-293 T) cells were stored in our laboratory. Detailed bacterial and cell culture conditions are detailed in a previous paper of the group [3].

### SS2 infection

The SS2 used for infection in this experiment was cultured to mid-log phase (OD_600_ of 0.4–0.6) at 37 °C, prewashed three times in phosphate buffer saline (PBS), and added to HCMEC/D3 cells at a multiplicity of infection (MOI) of 20. After stimulation at the indicated time points (0 h, 1 h, 2 h and 2.5 h), cells were used for subsequent experiments.

### *Ca*^*2*+^*effect determination*

Whether Ca^2+^ promoted RPSA protein aggregation or increased the effect of ENO protein on HCMEC/D3 cells was determined by titrating final CaCl_2_ (Aladdin, China) concentrations from 0 mM to 0.4 mM. Briefly, after cells were added to serum-free culture medium with or without CaCl_2_ at the additional indicated final concentration, the purified ENO protein was used to stimulate cells for indicated time. The concentration of CaCl_2_ used in this study is indicated in the relevant figure legends.

### Biomolecular condensate inhibition

1,6-Hexanediol (1,6-Hex) (Sigma-Aldrich, USA) was used as a protein aggregation inhibitor to destroy biomolecular condensates driven by IDR. Briefly, cells were treated with or without 1,6-Hex for 10 min and used for subsequent experiments. 1,6-Hex was applied to HEK-293 T cells at a dose of 5% and to HCMEC/D3 cells at a concentration of 2.5%.

### Cell transfection

HEK-293 T cells were collected, divided into 12-well plates (Corning Inc., USA) in 1 mL of complete growth medium unless otherwise indicated, and allowed to grow to 70–80% confluence. Transfection of the DNA plasmids (see Additional file 2: Table S1 for details) was performed with X-tremeGENE HP DNA transfection reagent (Roche, USA) according to the manufacturer’s instructions. Briefly, 3 µL of transfection reagent and 1.0 µg indicated protein-expressing plasmid or 1.0 µg shRNA-expressing plasmid formed a DNA-lipid complex in a serum-free DMEM/F12 medium (Gibco USA). When two different plasmids need to be co-transfected into HEK-293 T cells, the dosage of each plasmid is 0.5 ug. The DNA-lipid complexes were then added to the prepared HEK-293 T cells after being incubated at room temperature for 20 min.

### *Western blotting* (WB)

Total proteins from the treated cells were extracted using RIPA lysis buffer (Solarbio Life Sciences, China). First, RIPA lysis buffer was added to cells on ice after removing the cell culture media. After centrifugation at ~ 13,000 g for 10 min, the cell supernatant was collected. Equal quantities of proteins were separated by SDS-PAGE and transferred to a PVDF membrane (Millipore, USA) after being solubilized in sodium dodecyl sulfate (SDS) sample buffer. The primary antibodies were applied to the membrane and incubated overnight at 4 °C. The ECL chemiluminescent imaging equipment (Tanon, China) was used to determine signal intensity after the membranes were treated with an HRP-conjugated secondary antibody at room temperature for ~ 1 h. The WB experiments were quantified using Image-J software (National Institutes of Health, USA). The commercial antibodies used in the current study were in Additional file 2: Table S3. Uncropped Western blots were in Additional file 3.

### RPSA pull down

HCMEC/D3 cells were used for this experiment, and three groups were set up, namely, no antibody added group, uninfected group and SS2 infected-group. After 1 h of SS2 infection, RPSA antibody was added to the uninfected group and the infected group, respectively. Pull-down experiments were then performed using the Pierce™ Classic Magnetic Bead Immunoprecipitation/Immunoprecipitation Kit (Thermo Scientific, USA). Finally, silver staining and mass spectrometry sequencing of these three groups were performed (QL Bio., China). The commercial antibody used in the current study was in Additional file 2: Table S3.

### *Co-immunoprecipitation* (CO-IP)

The cells were seeded into 6-well plates (Corning Inc., USA) in 2 mL of complete growth medium. After 12 h, 2.0 µg of plasmids expressing two different indicated proteins were co-transfected into HEK-293 T cells in equal proportions. After transfection, the cells were further incubated for the indicated times (see the relevant figure legends for details). Subsequently, a CO-IP experiment was performed using the Pierce™ Classic Magnetic Bead Immunoprecipitation/Immunoprecipitation Kit (Thermo Scientific, USA). In brief, total proteins were extracted by adding ice-cold IP Lysis/Wash buffer, and after centrifugation at ~ 13,000 g for 10 min, the collected cell lysate was incubated with the indicated IP antibody at 4 ◦C overnight to form an antigen/antibody complex. Next, the complex was combined with protein A/G magnetic beads at room temperature for 1 h. Finally, after washing three times ultrapure water, the complex was analyzed by WB. The commercial antibody used in the current study was in Additional file 2: Table S3.

### *Immunofluorescence staining* (IF)

Cells were washed and fixed with 4% paraformaldehyde for 15 min at room temperature, and permeabilized for 15 min with 0.4% Triton X-100 (Solarbio, China). The step of cell permeabilization was skipped in cases where proteins on the cell surface were being analyzed. In addition, this step was also skipped when observing the indicated proteins carrying fluorescent tags in HEK-293 T cells. After the cells were blocked in normal goat serum (Solarbio, China) for 1 h, the samples were incubated overnight with the indicated primary antibodies at 4 ◦C. Finally, after the samples were incubated with secondary antibodies and were stained with Hoechst 33,342 dye (Sigma-Aldrich, USA), cells were imaged using fluorescence microscopy and confocal microscopy (Olympus, Japan). Please refer to our previous paper for data analyses, including selection of fluorescent regions, analysis of the mean fluorescence intensity, and analysis of fluorescence co-localization [3]. The commercial antibodies used in the current study are listed in Additional file 2: Table S3.

### RPSA protein production capacity determination

HEK-293 T cells were transfected with the indicated plasmids expressing EGFP-RPSA^WT^ or its mutant proteins. After transfection, the cells were further incubated for 24 h and washed three times. Subsequently, we used the 0.25% trypsin solution without EDTA (Beyotime, China) to harvest the cell. The cell suspension in each well was collected, centrifuged, and resuspended in PBS. Finally, the mean fluorescence intensity was analyzed by flow cytometry (BD Biosciences, USA). Quantification was performed by FlowJo (BD Life Sciences). The protein production level was also detected by western blotting using the antibody against GFP. Quantification was performed by Image J (National Institutes of Health, USA). The commercial antibodies used in the current study are listed in Additional file 2: Table S3.

### Bioinformatics analysis

The secondary structure of the RPSA protein was analyzed using online software (https://www.novopro.cn/). Data visualization of protein abundance from mass spectrometry analysis in the form of a heat-map was by using the Multiple Experiment Viewer (MEV) software (http://mev.tm4.org/) [28]. Gene Ontology (GO) biological pathway (BP) analysis was performed by using Metascape online software (http://metascape.org/) [28]. RPSA IDR sequence domains were predicted using the online software IUPred2A (https://iupred2a.elte.hu/) [29]. The Alphafold Protein Structure Database (https://alphafold.ebi.ac.uk/) was used to analyze the tertiary structure of the RPSA protein [30, 31].

### Multiples fluorescence immunohistochemistry (mIHC)

A multiplex fluorescent immunohistochemical staining kit (Absin, China) was used to perform multicolor fluorescent labeling on piglet brain tissue sample which were obtained from our previous study [5]. Akoya Mantra/Vectra/Polaris imaging equipment (Akoya Bioscienc, USA) was used, and images taken according to the dye spectrum conditions. Finally, the stained tissue slices were imaged. Quantitative analysis of images used HALO software (Indica Labs, USA). The specific experimental procedure follows the manufacturer’s instructions and the commercial antibodies used are listed in Additional file 2: Table S3.

### *Droplet formation assay *in vitro

Titration with a final protein concentration of 5 mM purified EGFP, EGFP-ENO and EGFP-RPSA proteins were diluted from storage buffer into droplet formation buffer (20 mM HEPES, pH 7.4, 2% PEG-8000). Titration with a final Ca^2+^ concentration of 3 mM allowed estimation of the effect of Ca^2+^ on the formation of concentrates from individual proteins. All groups were incubated at 37 °C for 10 min. Formation of condensates was observed by bright field and fluorescence microscopy using a 100 × oil objective. The ability of proteins to form concentrates was quantified by measuring the fluorescence size of the droplets. The average size of droplets in vitro was quantified using Image J (National Institutes of Health, USA).

### FRAP imaging and analysis

FRAP experiments were performed on an inverted confocal microscope (Olympus, Japan). Images were acquired using a × 100 oil-immersion objective, and the microscope was controlled using Olympus FV315-SW software. In FRAP experiments, 60 frames were acquired at a frame rate of 3 s, allowing 3 frames to be acquired before photobleaching. A bleach spot was chosen in a region of interest (ROI) at an aggregated fluorescence position. Condensates were fully or partially photobleached with 100% laser power for ~ 1 s. Pre-bleach and post-bleach images were acquired with a 490 nm laser. Representative FRAP images of condensates were taken before and after photobleaching at the indicated times. "CI" denotes a continuous imaging combination, recording at an average frequency of once every 3 s for 3 min. "Bleached" denotes the extent of fluorescence quenching, and subsequent continuous shooting is used to measure the extent of fluorescence recovery. Fluorescence intensities of ROIs were corrected using unbleached control regions and then normalized to the pre-bleached intensities of the ROIs. The mean fluorescence intensity of each group was analyzed by Image J (National Institutes of Health, USA). FRAP data were performed using GraphPad Prism 8 (GraphPad Prism Software Inc., USA).

### Mitochondrial activity assay

The purified ENO protein was added to HCMEC/D3 cells which were pretreated with serum-free culture medium at a concentration of 30 µg/mL. After stimulation for 0 h, 12 h and 24 h, 200 nM Mito-Tracker Red (Beyotime, China) working solution was added to each group. After incubation at 37 °C in the dark for 30 min, the samples were washed twice with D-Hank’s buffer. Finally, images were collected using a fluorescence microscope.

### *Ca*^*2*+^*concentration assays*

For intracellular Ca^2+^ concentration assay, a working solution of 0.5 μM Fluo-4AM (Beyotime, China) was added to each group cells incubated for 30 min. Similarly, a final concentration of 1 μM Rhod2-AM probe was added to each group to detect mitochondrial Ca^2+^ content. After incubation at 37 °C in the dark for 35 min, cell samples were also washed twice with D-Hank’s buffer. Finally, cells were harvested by trypsinization (no EDTA) and fluorescence units determined using a fluorescence microplate reader (SpectraMax iD3, USA).

### *Mitochondria potential and* reactive oxygen species (*ROS) assays*

For mitochondria potential assay, a working solution of JC-1 probe (1 ×) (Beyotime, China) was added to each group and cells incubated for 20 min in the dark. For ROS assay, a working solution of 10 µM DCFH-DA probe (Beyotime, China) was added to each group and cells incubated for 30 min in the dark. Fluorescence units were determined using a fluorescence microplate reader (SpectraMax iD3, USA).

### Blocking antibody and gene interference

ENO-positive and negative serum was incubated at 50 °C for 30 min, mixed with bacteria were mixed in a ratio of 1:1, and stimulated for 2 h. The level of cell mitochondrial membrane potential, ROS and Ca^2+^ content was measured.

RPSA and VIM interference plasmids used in this study were designed and synthesized by the Public Protein/Plasmid Library (PPL). Their target sequences are shown in Additional file 2: Table S2.

### *Mitochondrial protective agent and Ca*^*2*+^*chelating agent experiments*

ENO (30 µg/mL) was used to stimulate HCMEC/D3 cells for 12 h. Next, after three PBS washes, a final concentration of 10 µM mitochondrial protective agent MTP-131 (Selleck, USA) was added to the cells. After 40 min, fresh serum-free cell culture medium was added as replacement medium and cells incubated for a further 12 h. Similarly, a final concentration of 50 µM Ca^2+^ chelating agent EGTA-AM (MCE, USA) was used for 30 min to inhibit intracellular Ca^2+^.

### Apoptosis experiments

An Annexin V-FITC/PI apoptosis detection kit (Beyotime, China) was used to detect cellular apoptosis. In brief, after the cells were washing 3 times and processed by trypsin digestion (no EDTA), the cell suspension in each well was collected, centrifuged and resuspended in Annexin V-FITC binding solution. Annexin V-FITC and propidium iodide (PI) staining solution was added to each sample and incubated at room temperature for 15 min. Finally, cells were detected using a flow cytometer (BD Biosciences, USA). The ratio of dead cells to total cells in the indicated conditions was analyzed by FlowJo (BD Life Sciences).

### Statistical analyses

Statistical analysis and Bar graphs were performed using GraphPad Prism 8 software (GraphPad, Prism Software Inc., USA). Statistical significance calculations comparing two conditions were performed using a two-tailed unpaired Student’s t-test. Three or more sets of data were analyzed by one-way analysis of variance (ANOVA) with Tukey’s test. Experimental data are presented as mean ± SD. The Shapiro–Wilk test was used to determine if data were distributed normally. Data that were not normally distributed, are presented as mean ± median. In this case, the Mann–Whitney test was used for differentiation analysis among two groups. Three or more sets of data were analyzed by one-way ANOVA with Kruskal–Wallis test. Data are representative of at least three experiments. Sample numbers (e.g., number of cells or condensates) are indicated in the relevant figure legends, and individual data points are listed in Additional file 4. Statistical significance levels are denoted as follows: NS, not significant; *P < 0.05; **P < 0.01; ***P < 0.001.

### Supplementary Information

**Additional file 1: Fig. S1.** RPSA forms spherical condensations. (A) By use of appropriate antibodies in an immunofluorescence experiment, RPSA condensates are shown to localize in HCMEC/D3 cells. A representative image is displayed (scale bar = 40 μm) and magnified. (B) Representative confocal images of RPSA condensates formed by in vitro reconstitution using purified EGFP-RPSAWT. EGFP-ENO was used as a control. A representative image is displayed (scale bar = 10 μm) and magnified (scale bar = 5 μm). **Fig. S2.** RPSA is associated with intermediate filament-related proteins. (A) After SS2 infection for 1 h, cell lysates were used for pull-down analysis by using antibody against RPSA, followed by SDS-PAGE and silver staining analysis. (B) The strips obtained from result (A) were sent for mass spectrometry sequencing and quantification (QL Bio, Beijing). Data visualization of protein abundance was performed by a heat map (http://mev.tm4.org/). (C) Proteins from result (B) were subjected to a Gene Ontology (GO) biological pathway (BP) analysis using the online Metascape software (http://metascape.org/). **Fig. S**3. Multiple fluorescence immunohistochemistry analyses of RPSA and VIM proteins from brain tissue of piglets. Changes in RPSA and VIM expression levels in piglet brain tissues before and after SS2 infection. The brain tissue is labeled with the indicated antibodies (scale bar = 3 mm). Quantitative analysis of images by using HALO software. **Fig. S4.** SS2 infection or ENO stimulation can damage host cell mitochondria. (A) After SS2 infection of HCMCE/D3 cells for the indicated times, VIM and mitochondria were observed by immunofluorescence using the antibodies against VIM and UQCRC1. (B) Representative confocal images are shown (scale bar = 40 μm) and magnified (scale bar = 20 μm). HCMEC/D3 cells were stimulated for the indicated times using the indicated final concentration of ENO protein. Mitochondrial activity was detected and analyzed by immunofluorescence. Representative images are shown (scale bar = 50 μm). (C and D) The indicated serum and SS2 were mixed and added together to the HCMEC/D3 cells. After 2 h, mitochondria potential (C) or reactive oxygen species level (D) was then detected. Data represent the mean ± SD (n = 4 biologically independent samples). NS for not significant, * for *P* < 0.05, *** for *P* < 0.001; one-way ANOVA with Tukey’s test. Fig. S5. Ca2+ promotes ENO to induce apoptosis. (A) Cells were stimulated for the indicated time using a final concentration of 30 μg/mL of ENO protein. Flow cytometry analysis of the apoptosis level of cells. In the specified circumstances, the ratio of dead cells to total cells was examined by FlowJo. (B) The HCMEC/D3 cells were given a final concentration of 200 μM Ca2+, 30 μg/mL ENO, or a combination of the two. After 12 h, flow cytometry was used to analyze the death level of cells. The ratio of dead to total cells in the indicated conditions was quantitatively analyzed by FlowJo as mean ± SD (n ≥ 2 biologically independent samples). NS for not significant, ** for *P* < 0.01; one-way ANOVA with Tukey’s test

**Additional file 2: Table S1.** Plasmids used in this study. **Table S2.** Oligonucleotides used in this study. **Table S3.** List of information about antibodies used in this study

**Additional file 3. **Raw data for western blots. Uncropped membranes are included as raw data.

**Additional file 4. **Supporting data values for figures. All data generated or analyzed during this study are included in this published article.

## Data Availability

All data generated or analyzed during this study are included in this published article and its supplementary information files. Images generated and analyzed in this study are either shown in the article or are included in Additional file 1. Information on plasmids, primers, and antibodies used in this study is shown in Additional file 2. All full membrane images for western blot studies are shown in Additional file 3. Raw data values for all figures are provided in Additional file 4. All data and materials of this study are available from the corresponding author upon reasonable request.

## Abbreviations

SS2: *Streptococcus suis* Serotype 2
ENO: Enolase
RPSA: Ribosomal protein SA
VIM: Vimentin
LAMR1: Laminin receptor 1
HBMECs: Human brain microvascular endothelial cells
HCMEC/D3: Human cerebral microvascular endothelial cell line
HEK-293 T: Human embryonic kidney 293 T
IDR: Internally disordered region
IDP: Intrinsically disordered protein
LLPS: Liquid–liquid phase separation
LCD: Low complexity domain
AA: Amino acid
D: Aspartic acid, Asp
E: Glutamic acid, Glu
K: Lysine, Lys
Q: Glutamine, Gln
A: Alanine, Ala
BBB: Blood–brain barrier
MMP: Mitochondrial membrane potential
ROS: Reactive oxygen species
FRAP: Fluorescence recovery after photobleaching
IF: Immunofluorescence
WB: Western blotting
CO-IP: Co-immunoprecipitation
mIHC: Multicolor fluorescence immunohistochemistry
MnIx: Mean fluorescence intensity
MOI: Multiplicity of infection
ROI: Region of interest
1,6-Hex: 1,6-Hexanediol
Ca^2+^: Calcium ions
LB: Luria Broth
GO: Gene Ontology
BP: Biological pathway
