# Supplementary figures and images for: Enolase of Streptococcus suis serotype 2 promotes biomolecular condensation of ribosomal protein SA for HBMECs apoptosis
Source: BMC Biol. 2024 Feb 8;22:33. doi: 10.1186/s12915-024-01835-y (PMC10854124; doi:10.1186/s12915-024-01835-y)

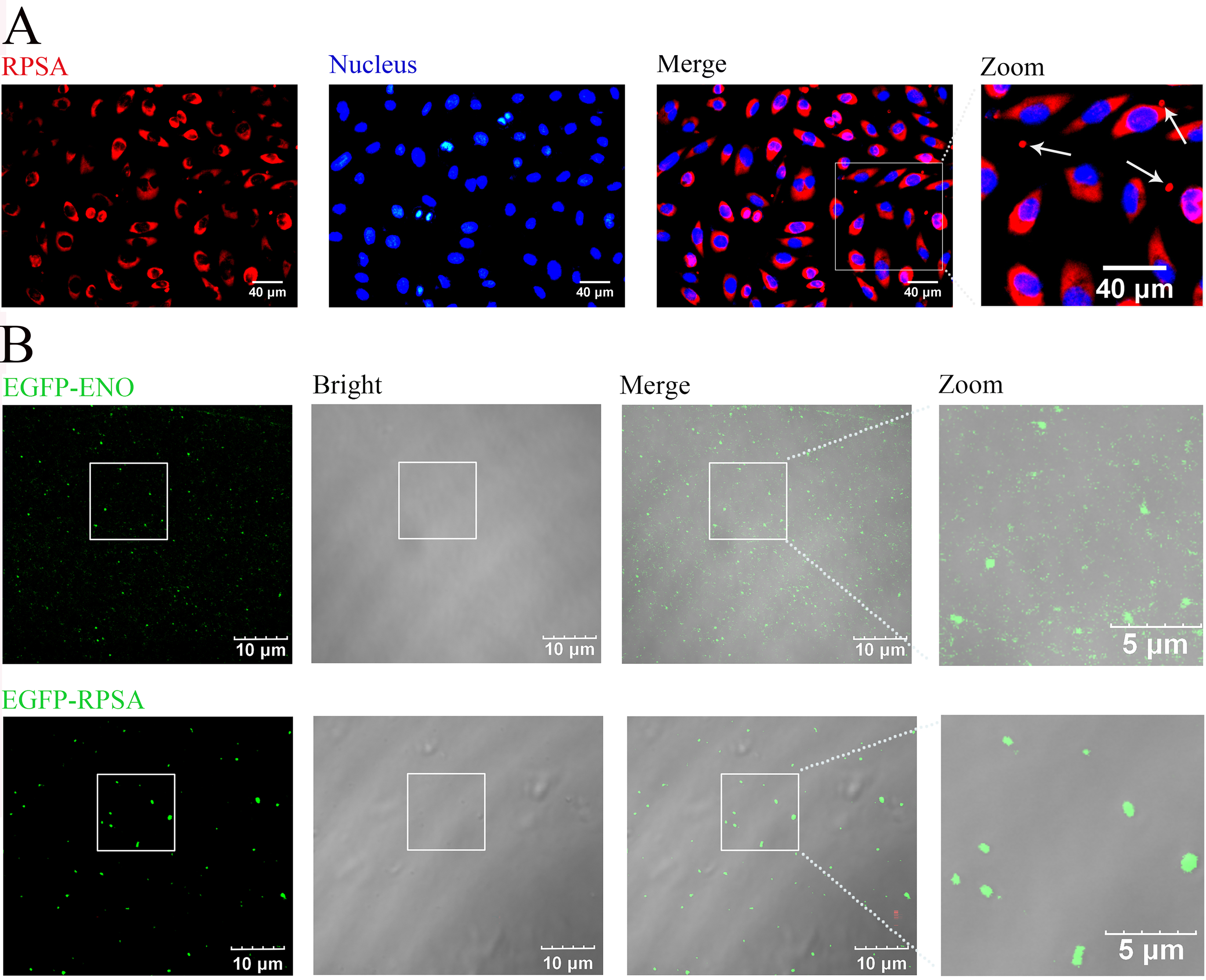

Supplement: Supplementary file 1 — Additional file 1: Fig. S1. RPSA forms spherical condensations. (A) By use of appropriate antibodies in an immunofluorescence experiment, RPSA condensates are shown to localize in HCMEC/D3 cells. A representative image is displayed (scale bar = 40 μm) and magnified. (B) Representative confocal images of RPSA condensates formed by in vitro reconstitution using purified EGFP-RPSAWT. EGFP-ENO was used as a control. A representative image is displayed (scale bar = 10 μm) and magnified (scale bar = 5 μm). Fig. S2. RPSA is associated with intermediate filament-related proteins. (A) After SS2 infection for 1 h, cell lysates were used for pull-down analysis by using antibody against RPSA, followed by SDS-PAGE and silver staining analysis. (B) The strips obtained from result (A) were sent for mass spectrometry sequencing and quantification (QL Bio, Beijing). Data visualization of protein abundance was performed by a heat map (http://mev.tm4.org/). (C) Proteins from result (B) were subjected to a Gene Ontology (GO) biological pathway (BP) analysis using the online Metascape software (http://metascape.org/). Fig. S3. Multiple fluorescence immunohistochemistry analyses of RPSA and VIM proteins from brain tissue of piglets. Changes in RPSA and VIM expression levels in piglet brain tissues before and after SS2 infection. The brain tissue is labeled with the indicated antibodies (scale bar = 3 mm). Quantitative analysis of images by using HALO software. Fig. S4. SS2 infection or ENO stimulation can damage host cell mitochondria. (A) After SS2 infection of HCMCE/D3 cells for the indicated times, VIM and mitochondria were observed by immunofluorescence using the antibodies against VIM and UQCRC1. (B) Representative confocal images are shown (scale bar = 40 μm) and magnified (scale bar = 20 μm). HCMEC/D3 cells were stimulated for the indicated times using the indicated final concentration of ENO protein. Mitochondrial activity was detected and analyzed by immunofluorescence. Represen [file 12915_2024_1835_MOESM1_ESM.zip › Additional file1_ Fig S1.jpg]

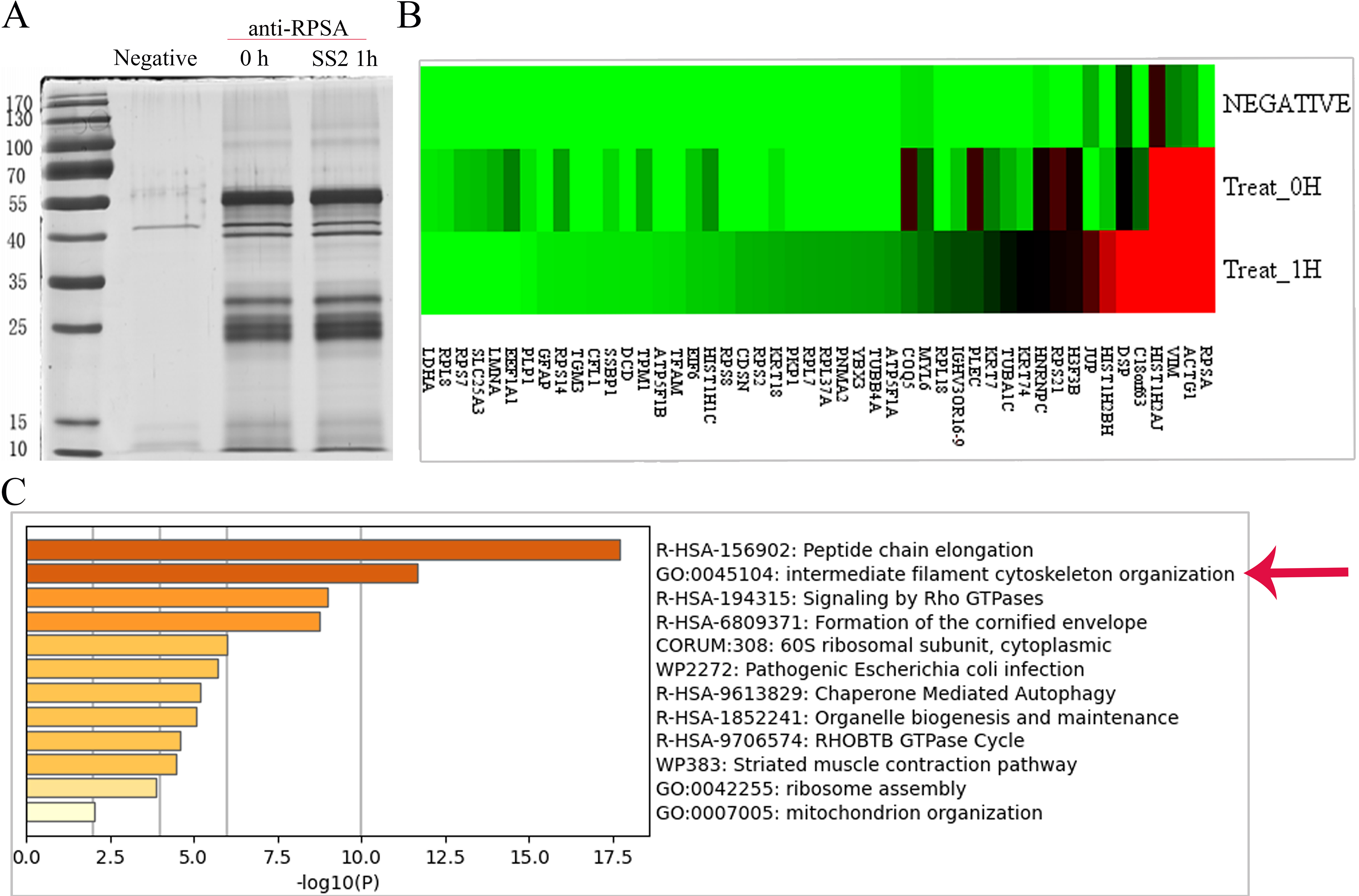

Supplement: Supplementary file 1 — Additional file 1: Fig. S1. RPSA forms spherical condensations. (A) By use of appropriate antibodies in an immunofluorescence experiment, RPSA condensates are shown to localize in HCMEC/D3 cells. A representative image is displayed (scale bar = 40 μm) and magnified. (B) Representative confocal images of RPSA condensates formed by in vitro reconstitution using purified EGFP-RPSAWT. EGFP-ENO was used as a control. A representative image is displayed (scale bar = 10 μm) and magnified (scale bar = 5 μm). Fig. S2. RPSA is associated with intermediate filament-related proteins. (A) After SS2 infection for 1 h, cell lysates were used for pull-down analysis by using antibody against RPSA, followed by SDS-PAGE and silver staining analysis. (B) The strips obtained from result (A) were sent for mass spectrometry sequencing and quantification (QL Bio, Beijing). Data visualization of protein abundance was performed by a heat map (http://mev.tm4.org/). (C) Proteins from result (B) were subjected to a Gene Ontology (GO) biological pathway (BP) analysis using the online Metascape software (http://metascape.org/). Fig. S3. Multiple fluorescence immunohistochemistry analyses of RPSA and VIM proteins from brain tissue of piglets. Changes in RPSA and VIM expression levels in piglet brain tissues before and after SS2 infection. The brain tissue is labeled with the indicated antibodies (scale bar = 3 mm). Quantitative analysis of images by using HALO software. Fig. S4. SS2 infection or ENO stimulation can damage host cell mitochondria. (A) After SS2 infection of HCMCE/D3 cells for the indicated times, VIM and mitochondria were observed by immunofluorescence using the antibodies against VIM and UQCRC1. (B) Representative confocal images are shown (scale bar = 40 μm) and magnified (scale bar = 20 μm). HCMEC/D3 cells were stimulated for the indicated times using the indicated final concentration of ENO protein. Mitochondrial activity was detected and analyzed by immunofluorescence. Represen [file 12915_2024_1835_MOESM1_ESM.zip › Additional file1_ Fig S2.jpg]

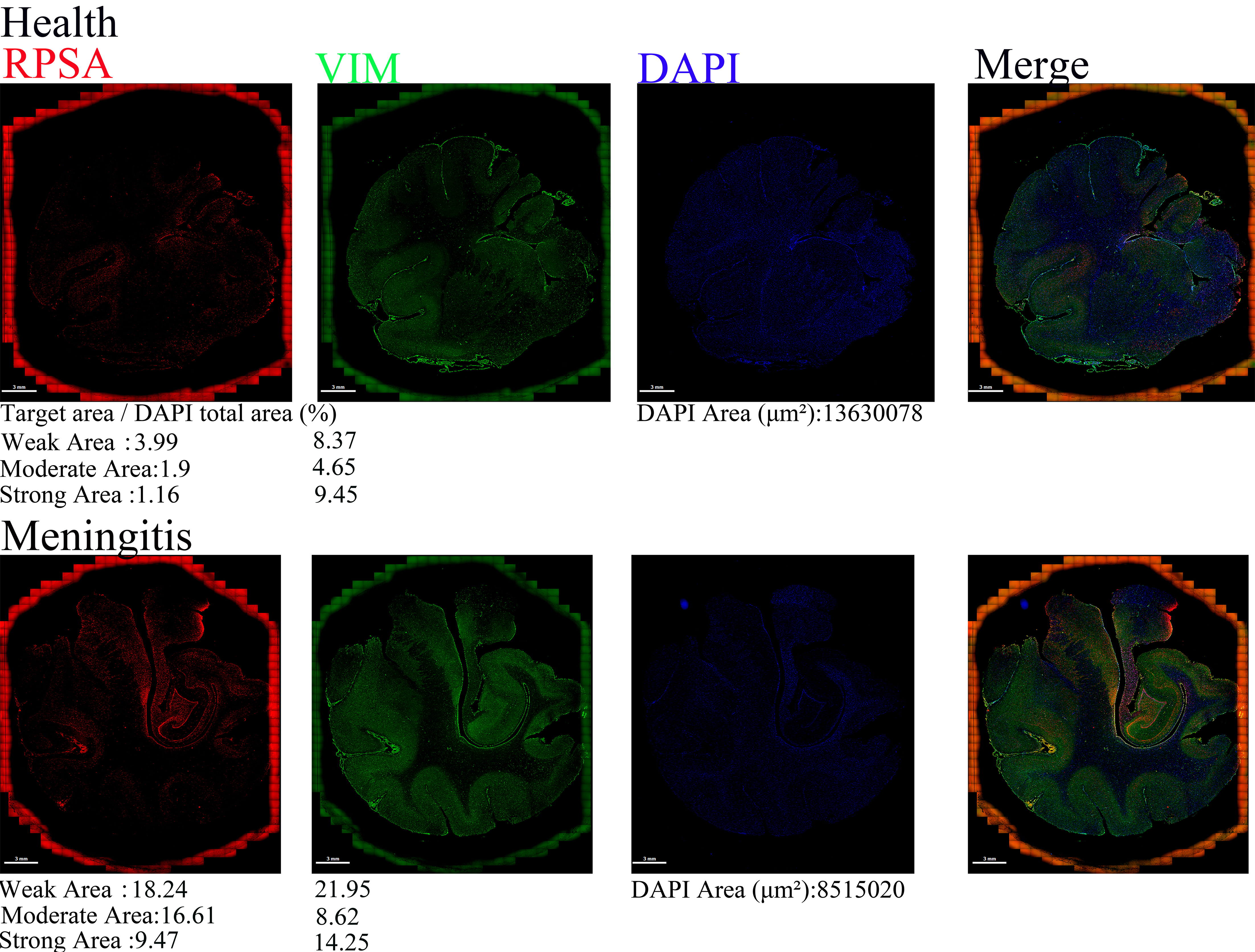

Supplement: Supplementary file 1 — Additional file 1: Fig. S1. RPSA forms spherical condensations. (A) By use of appropriate antibodies in an immunofluorescence experiment, RPSA condensates are shown to localize in HCMEC/D3 cells. A representative image is displayed (scale bar = 40 μm) and magnified. (B) Representative confocal images of RPSA condensates formed by in vitro reconstitution using purified EGFP-RPSAWT. EGFP-ENO was used as a control. A representative image is displayed (scale bar = 10 μm) and magnified (scale bar = 5 μm). Fig. S2. RPSA is associated with intermediate filament-related proteins. (A) After SS2 infection for 1 h, cell lysates were used for pull-down analysis by using antibody against RPSA, followed by SDS-PAGE and silver staining analysis. (B) The strips obtained from result (A) were sent for mass spectrometry sequencing and quantification (QL Bio, Beijing). Data visualization of protein abundance was performed by a heat map (http://mev.tm4.org/). (C) Proteins from result (B) were subjected to a Gene Ontology (GO) biological pathway (BP) analysis using the online Metascape software (http://metascape.org/). Fig. S3. Multiple fluorescence immunohistochemistry analyses of RPSA and VIM proteins from brain tissue of piglets. Changes in RPSA and VIM expression levels in piglet brain tissues before and after SS2 infection. The brain tissue is labeled with the indicated antibodies (scale bar = 3 mm). Quantitative analysis of images by using HALO software. Fig. S4. SS2 infection or ENO stimulation can damage host cell mitochondria. (A) After SS2 infection of HCMCE/D3 cells for the indicated times, VIM and mitochondria were observed by immunofluorescence using the antibodies against VIM and UQCRC1. (B) Representative confocal images are shown (scale bar = 40 μm) and magnified (scale bar = 20 μm). HCMEC/D3 cells were stimulated for the indicated times using the indicated final concentration of ENO protein. Mitochondrial activity was detected and analyzed by immunofluorescence. Represen [file 12915_2024_1835_MOESM1_ESM.zip › Additional file1_ Fig S3.jpg]

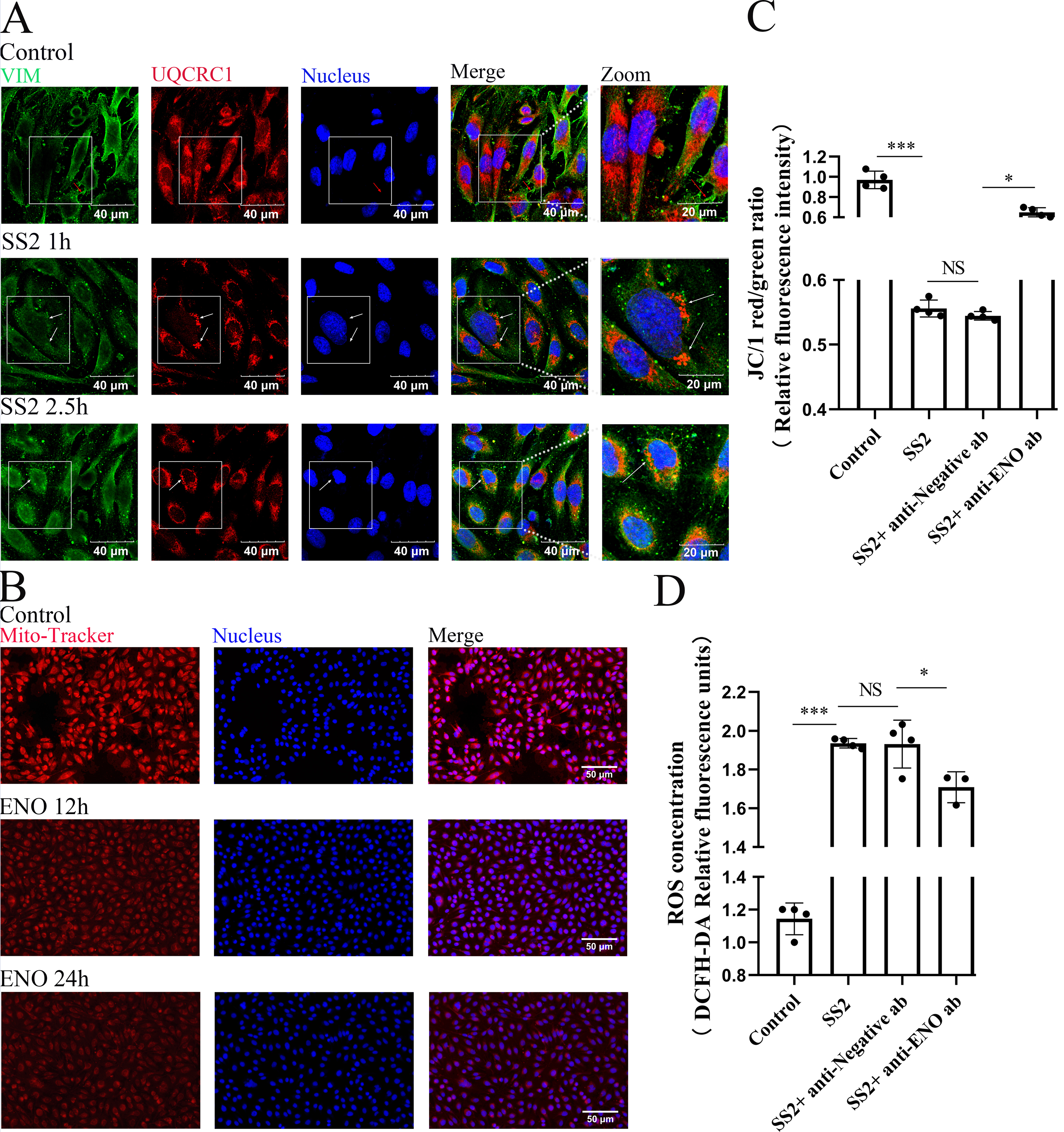

Supplement: Supplementary file 1 — Additional file 1: Fig. S1. RPSA forms spherical condensations. (A) By use of appropriate antibodies in an immunofluorescence experiment, RPSA condensates are shown to localize in HCMEC/D3 cells. A representative image is displayed (scale bar = 40 μm) and magnified. (B) Representative confocal images of RPSA condensates formed by in vitro reconstitution using purified EGFP-RPSAWT. EGFP-ENO was used as a control. A representative image is displayed (scale bar = 10 μm) and magnified (scale bar = 5 μm). Fig. S2. RPSA is associated with intermediate filament-related proteins. (A) After SS2 infection for 1 h, cell lysates were used for pull-down analysis by using antibody against RPSA, followed by SDS-PAGE and silver staining analysis. (B) The strips obtained from result (A) were sent for mass spectrometry sequencing and quantification (QL Bio, Beijing). Data visualization of protein abundance was performed by a heat map (http://mev.tm4.org/). (C) Proteins from result (B) were subjected to a Gene Ontology (GO) biological pathway (BP) analysis using the online Metascape software (http://metascape.org/). Fig. S3. Multiple fluorescence immunohistochemistry analyses of RPSA and VIM proteins from brain tissue of piglets. Changes in RPSA and VIM expression levels in piglet brain tissues before and after SS2 infection. The brain tissue is labeled with the indicated antibodies (scale bar = 3 mm). Quantitative analysis of images by using HALO software. Fig. S4. SS2 infection or ENO stimulation can damage host cell mitochondria. (A) After SS2 infection of HCMCE/D3 cells for the indicated times, VIM and mitochondria were observed by immunofluorescence using the antibodies against VIM and UQCRC1. (B) Representative confocal images are shown (scale bar = 40 μm) and magnified (scale bar = 20 μm). HCMEC/D3 cells were stimulated for the indicated times using the indicated final concentration of ENO protein. Mitochondrial activity was detected and analyzed by immunofluorescence. Represen [file 12915_2024_1835_MOESM1_ESM.zip › Additional file1_ Fig S4.jpg]

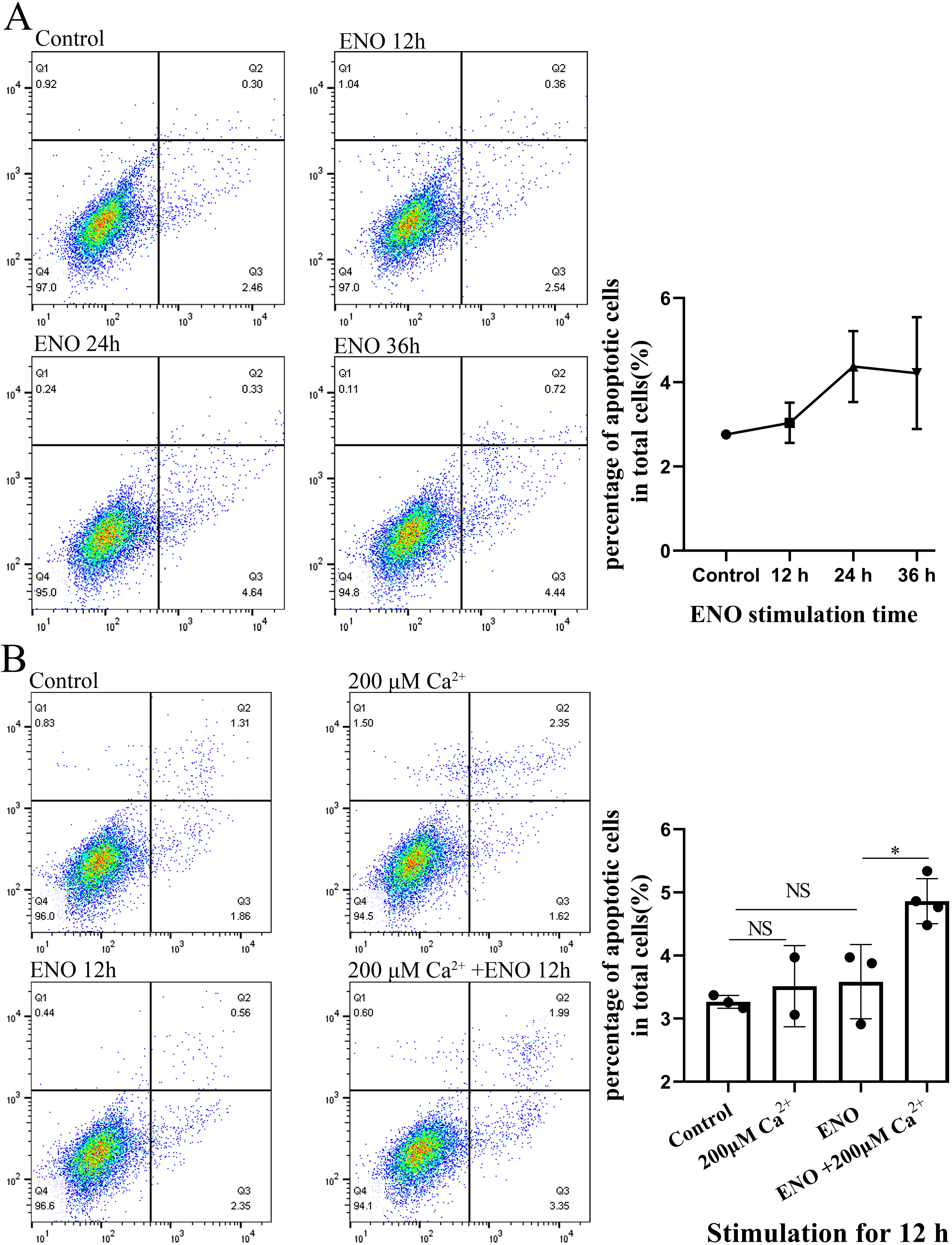

Supplement: Supplementary file 1 — Additional file 1: Fig. S1. RPSA forms spherical condensations. (A) By use of appropriate antibodies in an immunofluorescence experiment, RPSA condensates are shown to localize in HCMEC/D3 cells. A representative image is displayed (scale bar = 40 μm) and magnified. (B) Representative confocal images of RPSA condensates formed by in vitro reconstitution using purified EGFP-RPSAWT. EGFP-ENO was used as a control. A representative image is displayed (scale bar = 10 μm) and magnified (scale bar = 5 μm). Fig. S2. RPSA is associated with intermediate filament-related proteins. (A) After SS2 infection for 1 h, cell lysates were used for pull-down analysis by using antibody against RPSA, followed by SDS-PAGE and silver staining analysis. (B) The strips obtained from result (A) were sent for mass spectrometry sequencing and quantification (QL Bio, Beijing). Data visualization of protein abundance was performed by a heat map (http://mev.tm4.org/). (C) Proteins from result (B) were subjected to a Gene Ontology (GO) biological pathway (BP) analysis using the online Metascape software (http://metascape.org/). Fig. S3. Multiple fluorescence immunohistochemistry analyses of RPSA and VIM proteins from brain tissue of piglets. Changes in RPSA and VIM expression levels in piglet brain tissues before and after SS2 infection. The brain tissue is labeled with the indicated antibodies (scale bar = 3 mm). Quantitative analysis of images by using HALO software. Fig. S4. SS2 infection or ENO stimulation can damage host cell mitochondria. (A) After SS2 infection of HCMCE/D3 cells for the indicated times, VIM and mitochondria were observed by immunofluorescence using the antibodies against VIM and UQCRC1. (B) Representative confocal images are shown (scale bar = 40 μm) and magnified (scale bar = 20 μm). HCMEC/D3 cells were stimulated for the indicated times using the indicated final concentration of ENO protein. Mitochondrial activity was detected and analyzed by immunofluorescence. Represen [file 12915_2024_1835_MOESM1_ESM.zip › Additional file1_ Fig S5.jpg]
