# Supplementary material for: Enolase of Streptococcus suis serotype 2 promotes biomolecular condensation of ribosomal protein SA for HBMECs apoptosis
Source: BMC Biol. 2024 Feb 8;22:33. doi: 10.1186/s12915-024-01835-y (PMC10854124; doi:10.1186/s12915-024-01835-y)
Supplement: Supplementary file 2 — Additional file 2: Table S1. Plasmids used in this study. Table S2. Oligonucleotides used in this study. Table S3. List of information about antibodies used in this study [file 12915_2024_1835_MOESM2_ESM.zip › Additional file2_ Table S1.docx]

**Table S1. Plasmids were used in this study**

| Plasmids name | Description | Reference |
| --- | --- | --- |
| pET28a: ENO | Prokaryotic expression of ENO protein | Jiang H, et al., 2021 |
| pET28a: EGFP-ENO | Prokaryotic expression of EGFP-ENO protein | Jiang H, et al., 2021 |
| pET28a: EGFP-RPSA | Prokaryotic expression of EGFP-RPSA protein | This study |
| pMcherry-C1: ENO | For cell transfection validation of Co-IP and IF | This study |
| pEGFP-C1: RPSA | For cell transfection validation of Co-IP, IF and FRAP experiments | This study |
| p3*FLAG-CMV-9: VIM | For cell transfection to verify Co-IP experiments | This study |
| pEGFP-C1: RPSA_N1-206aa_ | For cell transfection validation of IF experiments and flow cytometry | This study |
| pEGFP-C1: RPSA_N1-228aa_ | For cell transfection validation of IF experiments and flow cytometry | This study |
| pEGFP-C1: RPSA_N1-264aa_ | For cell transfection validation of IF experiments and flow cytometry | This study |
| pEGFP-C1: RPSA_N1-206aa+IDR2_ | For cell transfection validation of IF experiments and flow cytometry | This study |
| pEGFP-C1: RPSA_N1-206aa+IDR3_ | For cell transfection validation of IF experiments and flow cytometry | This study |
| pEGFP-C1: RPSA_N1-206aa+IDR2+IDR3_ | For cell transfection validation of IF experiments and flow cytometry | This study |
| pEGFP-C1: RPSA_N1-206aa+IDR1+IDR3_ | For cell transfection validation of IF experiments and flow cytometry | This study |
| pEGFP-C1: RPSA_N1-206aa+IDR1(E/K)_ | For cell transfection validation of IF experiments flow cytometry and WB | This study |
| pEGFP-C1: RPSA_N1-206aa+IDR1(E/Q)_ | For cell transfection validation of IF experiments flow cytometry and WB | This study |
| pEGFP-C1: RPSA_N1-206aa+IDR1(E/A)_ | For cell transfection validation of IF experiments flow cytometry and WB | This study |
| pEGFP-C1: IDR | For cell transfection validation of Co-IP, IF and FRAP experiments | This study |
| pLKO.1-puro: shControl | Control group for target gene interference experiments | This study |
| pLKO.1-puro: shRPSA | Interference with *rpsa* gene expression after cell transfection | This study |
| pLKO.1-puro: shVIM | Interference with *vim* gene expression after cell transfection | This study |
