# Supplementary material for: Enolase of Streptococcus suis serotype 2 promotes biomolecular condensation of ribosomal protein SA for HBMECs apoptosis
Source: BMC Biol. 2024 Feb 8;22:33. doi: 10.1186/s12915-024-01835-y (PMC10854124; doi:10.1186/s12915-024-01835-y)
Supplement: Supplementary file 2 — Additional file 2: Table S1. Plasmids used in this study. Table S2. Oligonucleotides used in this study. Table S3. List of information about antibodies used in this study [file 12915_2024_1835_MOESM2_ESM.zip › Additional file2_ Table S2.docx]

**Table S2. Oligonucleotides used in this study**

| Primer name | Sequence (5^’^-3^’^) | |
| --- | --- | --- |
| Construction of pmcherry-C1: ENO | | |
| XhoI- ENO-F | CCGCTCGAGCTTCAATTATTACTGATG | |
| BamH1- ENO-R | CGCGGATCCTTATTTTTTCAAGTTGTAG | |
| Construction of pEGFP-C1: RPSA | | |
| XhoI- RPSA-F | CCGCTCGAGCTTCCGGAGCCCTTGATG | |
| EcoRI- RPSA-R | CCGGAATTCTTAAGACCAGTCAGTG | |
| Construction of p3*Flag-CMV-9: VIM | | |
| EcoR1- VIM-F | CCGGAATTCATCCACCAGGTCCGTGTC | |
| BamH1- VIM-R | CGCGGATCCTTATTCAAGGTCATCGT | |
| Construction of pEGFP-C1: RPSA_N1-206aa_ | | |
| XhoI- RPSA_1-206aa_-F | CCGCTCGAGCTTCCGGAGCCCTTGATG | |
| EcoRI- RPSA_1-206aa_-R | CCGGAATTCTTAATCTCTGTAGAAGTAC | |
| Construction of pEGFP-C1: RPSA_N1-228aa_ | | |
| XhoI- RPSA_1-206aa_-F | CCGCTCGAGCTTCCGGAGCCCTTGATG | |
| EcoRI- RPSA_1-228aa_-R | CCGGAATTCTTACTGAAATTCCTCCTTG | |
| Construction of pEGFP-C1: RPSA_N1-264aa_ | | |
| XhoI- RPSA_1-206aa_-F | CCGCTCGAGCTTCCGGAGCCCTTGATG | |
| EcoRI- RPSA_1-264aa_-R | CCGGAATTCTTAAGTAGGGAATTGCTGA | |
| Construction of: pEGFP-C1: RPSA_N1-206aa+IDR2_ | | |
| XhoI- upstream fragment -F | CCGCTCGAGCTTCCGGAGCCCTTGATG | |
| upstream fragment -R | CCATTCACCATCTCTGTAGAAGTA | |
| downstream fragment -F | CTACAGAGATGGTGAATGGACTGCT | |
| EcoRI- downstream fragment -R | CCGGAATTCTTAAGTAGGGAATTGCTGA | |
| Construction of: pEGFP-C1: RPSA_N1-206aa+IDR3_ | | |
| XhoI- upstream fragment -F | CCGCTCGAGCTTCCGGAGCCCTTGATG | |
| upstream fragment -R | TCCAGTCTTCATCTCTGTAGAAGTAC | |
| downstream fragment -F | CTACAGAGATGAAGACTGGAGCGCTC | |
| EcoRI- downstream fragment -R | CCGGAATTCTTAAGACCAGTCAGTG | |
| Construction of: pEGFP-C1: RPSA_N1-206aa+IDR2+IDR3_ | | |
| XhoI- upstream fragment -F | CCGCTCGAGCTTCCGGAGCCCTTGATG | |
| upstream fragment -R | CCATTCACCATCTCTGTAGAAGTA | |
| downstream fragment -F | CTACAGAGATGGTGAATGGACTGCT | |
| EcoRI- downstream fragment -R | CCGGAATTCTTAAGACCAGTCAGTGG | |
| Construction of: pEGFP-C1: RPSAN_1-206aa+IDR1+IDR3_ | | |
| XhoI- upstream fragment -F | CCGCTCGAGCTTCCGGAGCCCTTGATG | |
| upstream fragment -R | TCCAGTCTTCCTGAAATTCCTCCT | |
| downstream fragment -F | GAATTTCAGGAAGACTGGAGCGCT | |
| EcoRI- downstream fragment -R | CCGGAATTCTTAAGACCAGTCAGTGG | |
| Primer name | | Sequence (5^’^-3^’^) |
| Construction of: pEGFP-C1: RPSAN_1-206aa+IDR1 (EE/KK)_ | | |
| XhoI- upstream fragment -F | CCGCTCGAGCTTCCGGAGCCCTTGATG | |
| upstream fragment -R | AATCTTCTTAGGATCTCTGTAGAAG | |
| downstream fragment -F | CAGAGATCCTAAGAAGATTGAAAAA | |
| EcoRI- downstream fragment -R | CCGGAATTCTTACTGAAACTTCTTCTTGGT | |
| Construction of: pEGFP-C1: RPSAN_1-206aa+IDR1 (EE/QQ)_ | | |
| XhoI- upstream fragment -F | CCGCTCGAGCTTCCGGAGCCCTTGATG | |
| upstream fragment -R | AATCTGCTGAGGATCTCTGTAGAAG | |
| downstream fragment -F | CAGAGATCCTCAGCAGATTGAAAAAC | |
| EcoRI- downstream fragment -R | CCGGAATTCTTACTGAAACTGCTGCTTGGT | |
| Construction of: pEGFP-C1: RPSAN_1-206aa+IDR1 (EE/AA)_ | | |
| XhoI- upstream fragment -F | CCGCTCGAGCTTCCGGAGCCCTTGATG | |
| upstream fragment -R | ATAGCAGCAGGATCTCTGTAGAAG | |
| downstream fragment -F | CAGAGATCCTGCTGCTATTGAAAAAGC | |
| EcoRI- downstream fragment -R | CCGGAATTCTTACTGAAAAGCAGCCTTGGTC | |
| Construction of: pEGFP-C1: IDR | | |
| XhoI -IDR -F | CCGCTCGAGCTGAGATTGAAAAAGAAGA | |
| EcoRI -IDR -R | CCGGAATTCTTAAGACCAGTCAGTG | |
| Interfering plasmids | Primer sequences | |
| pLKO.1-puro: shControl | CCTAAGGTTAAGTCGCCCTCG | |
| pLKO.1-puro: shRPSA | CCTGCTGATGTCAGTGTTATA | |
| pLKO.1-puro:sh VIM | GCAAGTATCCAACCAACTT | |
