# Supplementary material for: Enolase of Streptococcus suis serotype 2 promotes biomolecular condensation of ribosomal protein SA for HBMECs apoptosis
Source: BMC Biol. 2024 Feb 8;22:33. doi: 10.1186/s12915-024-01835-y (PMC10854124; doi:10.1186/s12915-024-01835-y)
Supplement: Supplementary file 2 — Additional file 2: Table S1. Plasmids used in this study. Table S2. Oligonucleotides used in this study. Table S3. List of information about antibodies used in this study [file 12915_2024_1835_MOESM2_ESM.zip › Additional file2_ Table S3.docx]

**Table S3. List of information about antibodies used in this study**

| Product Name | Application | CAT. | Source | RRID |
| --- | --- | --- | --- | --- |
| RPSA antibody | IF (1:100); WB (1:2000); Pull down (1:50); | 14533-1-AP | Proteintech | AB_2182528 |
|  | mIHC (1:500) | NBP1-33002 | NOVUS | AB_2182396 |
| Vimentin antibody | IF (1:100); WB (1:5000); | 60330-1-Ig | Proteintech | AB_2881439 |
|  | mIHC (1:400) | abs131996 | Absin | AB_3073985 |
| UQCRC1 antibody | IF (1:100) | HPA002815 | Sigma-Aldrich | AB_1080486 |
| Alexa Fluor 488 Conjugate antibody | IF (1:800) | 4408 | CST | AB_10694704 |
| Alexa Fluor 594 Conjugate antibody | IF (1:800) | 8889 | CST | AB_2716249 |
| FLAG antibody | WB (1:1000) | AE004 | ABclonal | AB_2771921 |
|  | IP (1:100) | 66008-4-Ig | Proteintech | AB_2918475 |
| mCherry antibody | WB (1:2000) | 26765-1-AP | Proteintech | AB_2876881 |
| GFP antibody | WB (1:1000) | AE011 | ABclonal | AB_2771922 |
|  | IP (1:200) | 66002-1-Ig | Proteintech | AB_11182611 |
| 594-conjugated GFP antibody | IF (1:200) | CL594-66002 | Proteintech | AB_2919937 |
| GAPDH antibody | WB (1:5000) | 10494-1-AP | Proteintech | AB_2263076 |
| ENO -positive serum | blocking antibody | LEI_ENO | Liu H, et al., 2021 | AB_3073986 |
