# Supplementary material for: Enolase of Streptococcus suis serotype 2 promotes biomolecular condensation of ribosomal protein SA for HBMECs apoptosis
Source: BMC Biol. 2024 Feb 8;22:33. doi: 10.1186/s12915-024-01835-y (PMC10854124; doi:10.1186/s12915-024-01835-y)
Supplement: Supplementary file 3 — Additional file 3. Raw data for western blots. Uncropped membranes are included as raw data. [file 12915_2024_1835_MOESM3_ESM.docx]

**Additional file3_ Raw data for western blots.**

Maker


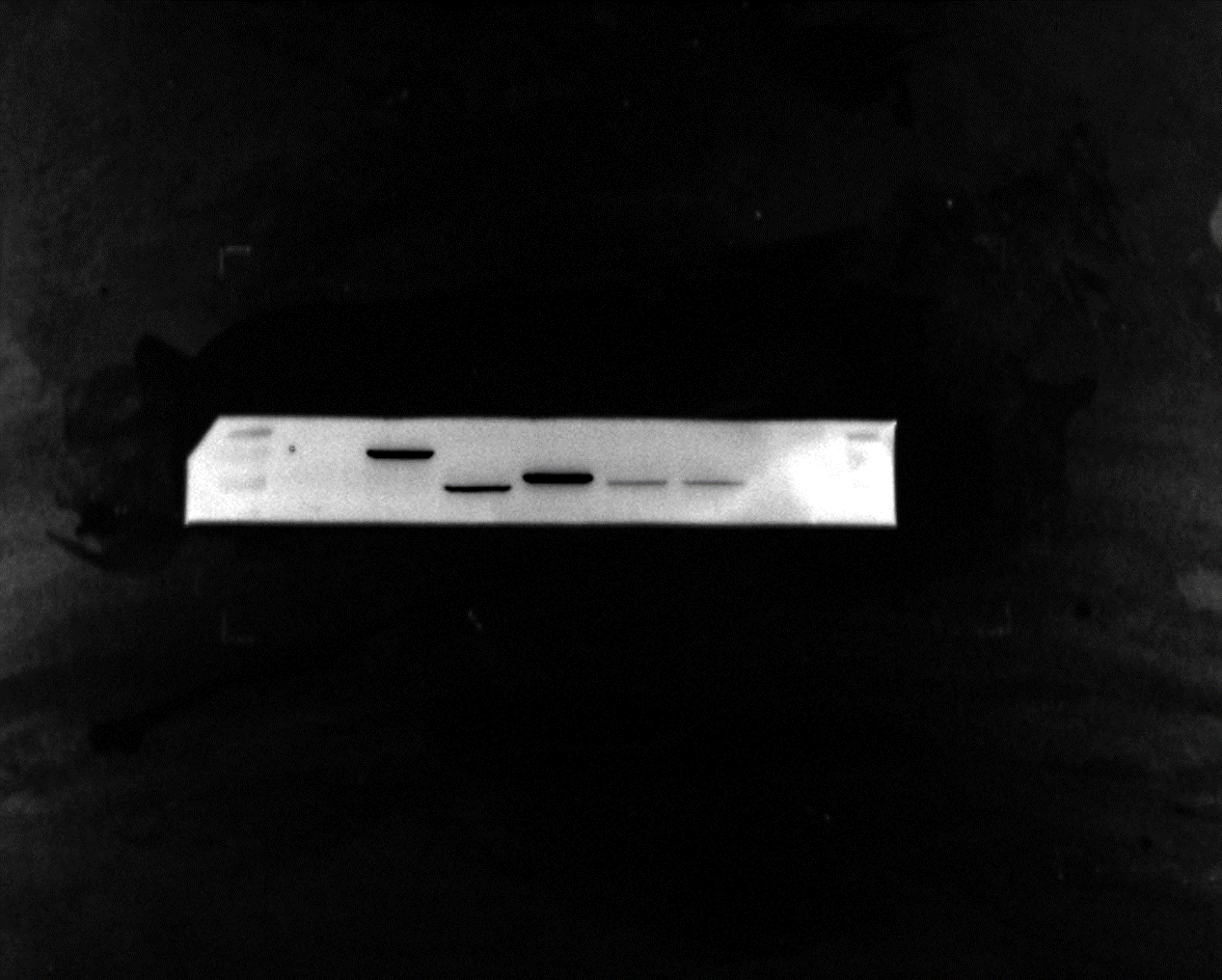


WB: GFP





WB: GAPDH

**Fig 3D Uncropped blot**

Maker


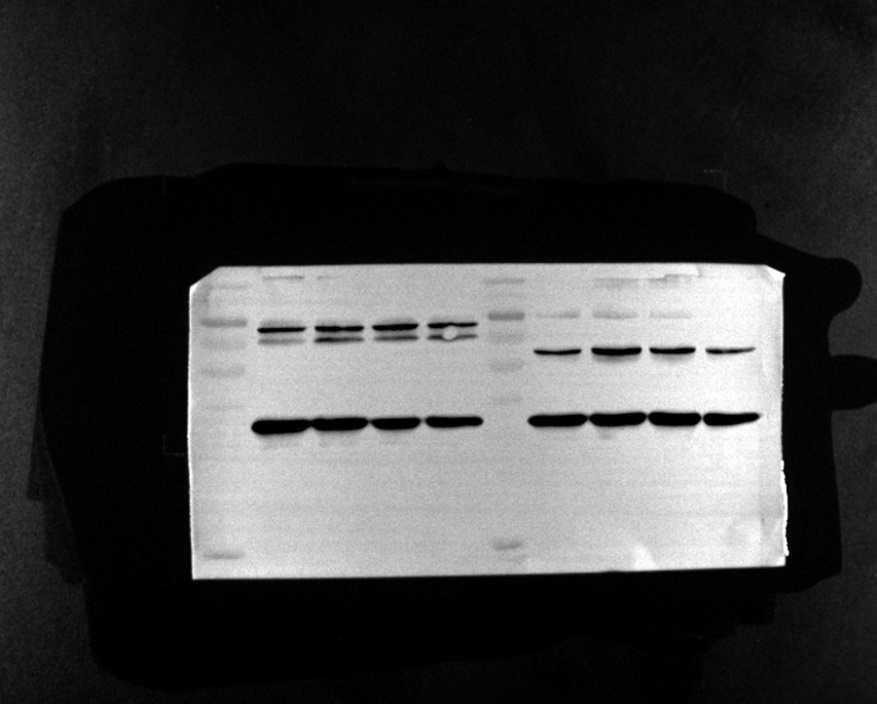


Input: mcherry-eno

WB: Mcherry (red box)

WB: GAPDH


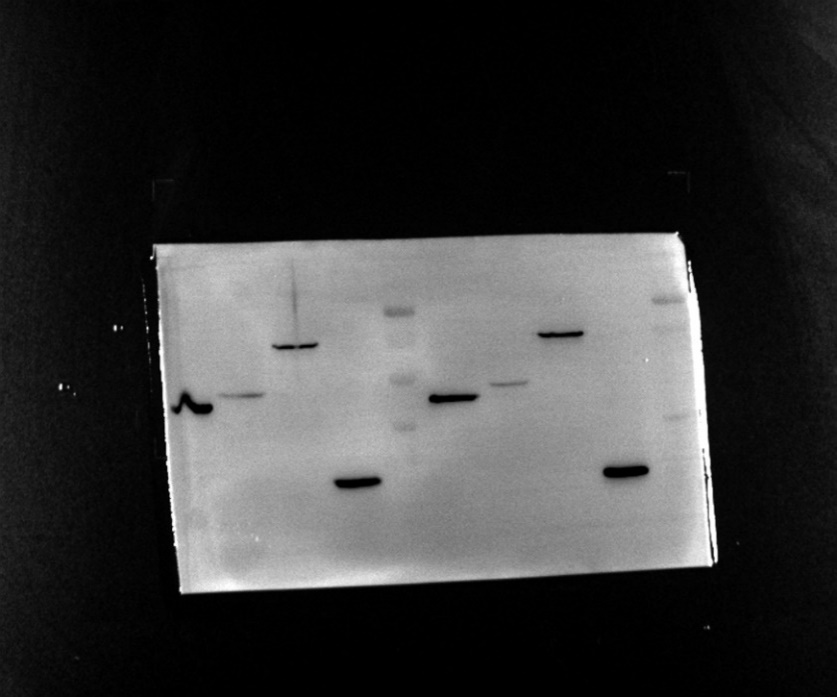


Input: GFP, GFP-RPSA-F/N/IDR

WB: GFP


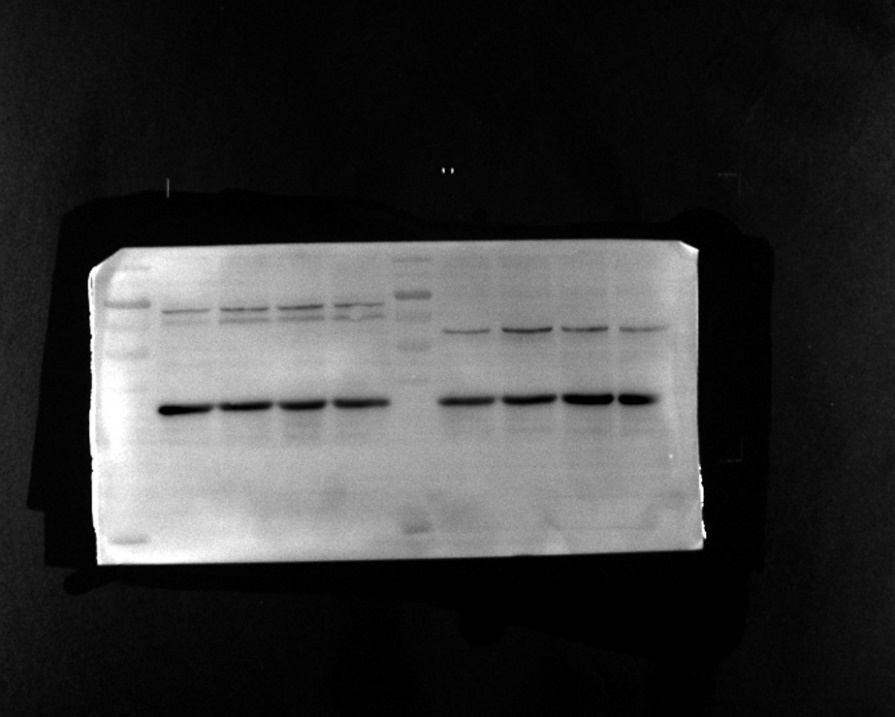


Input: mcherry-eno

WB: Mcherry

WB: GAPDH (red box)


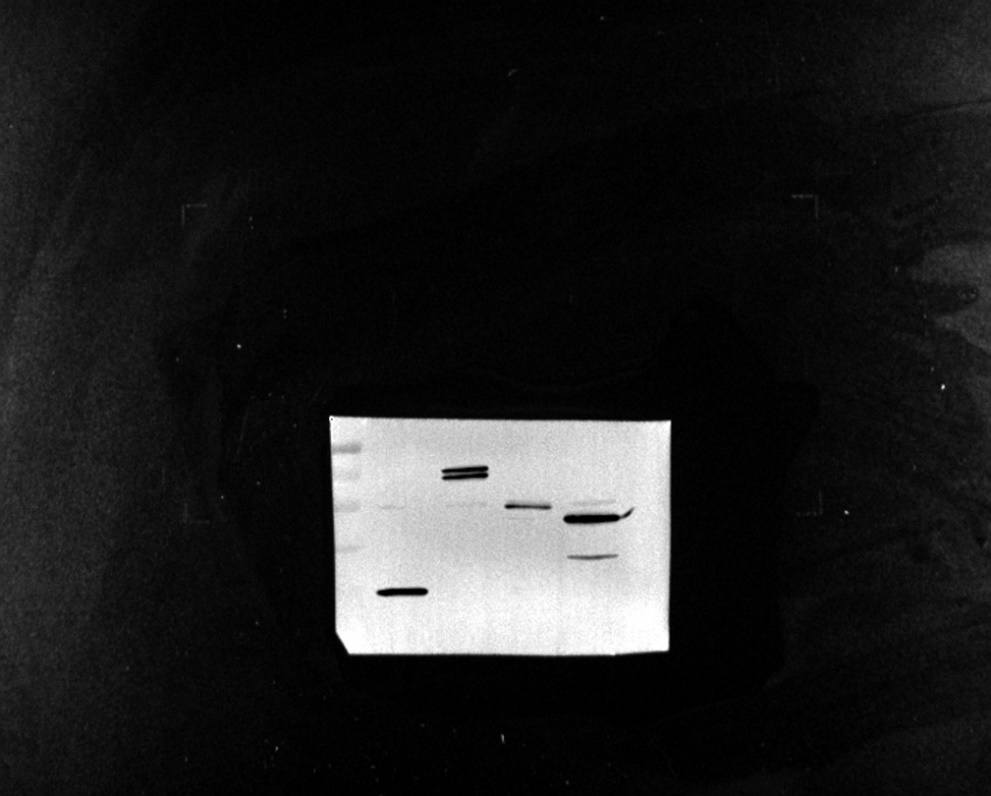


IP: GFP;

WB: GFP





IP: GFP;

WB: Mcherry (red box)

**Fig 5A Uncropped blot**

Maker


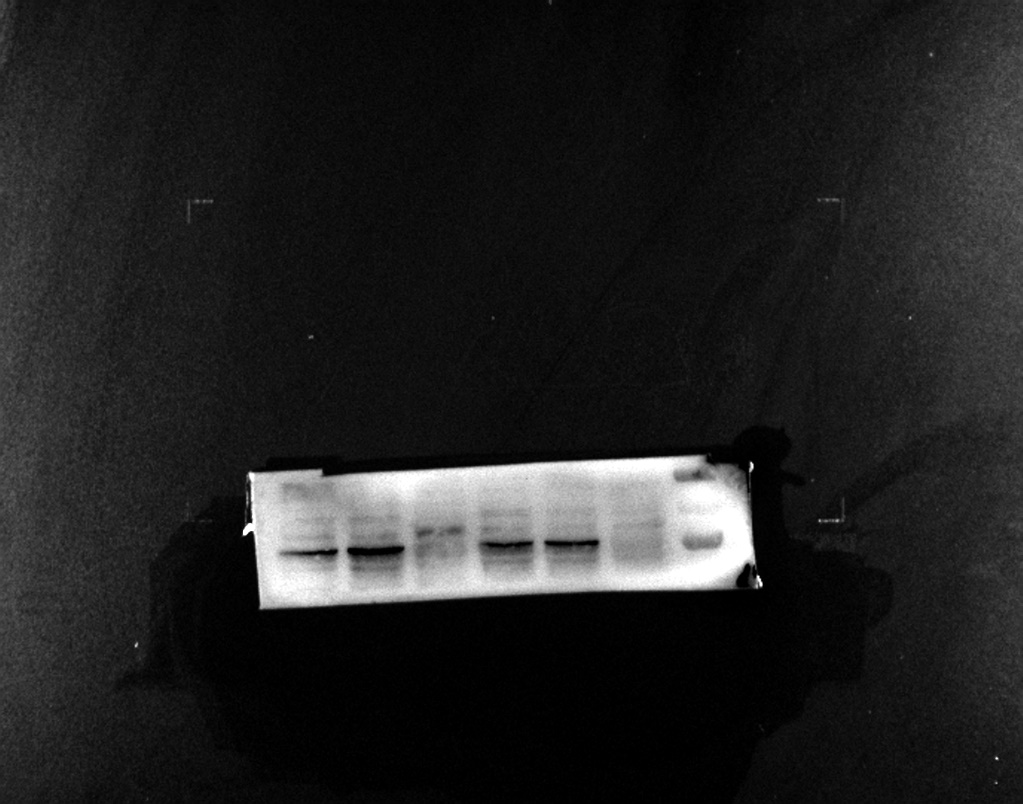


WB: His (red box)


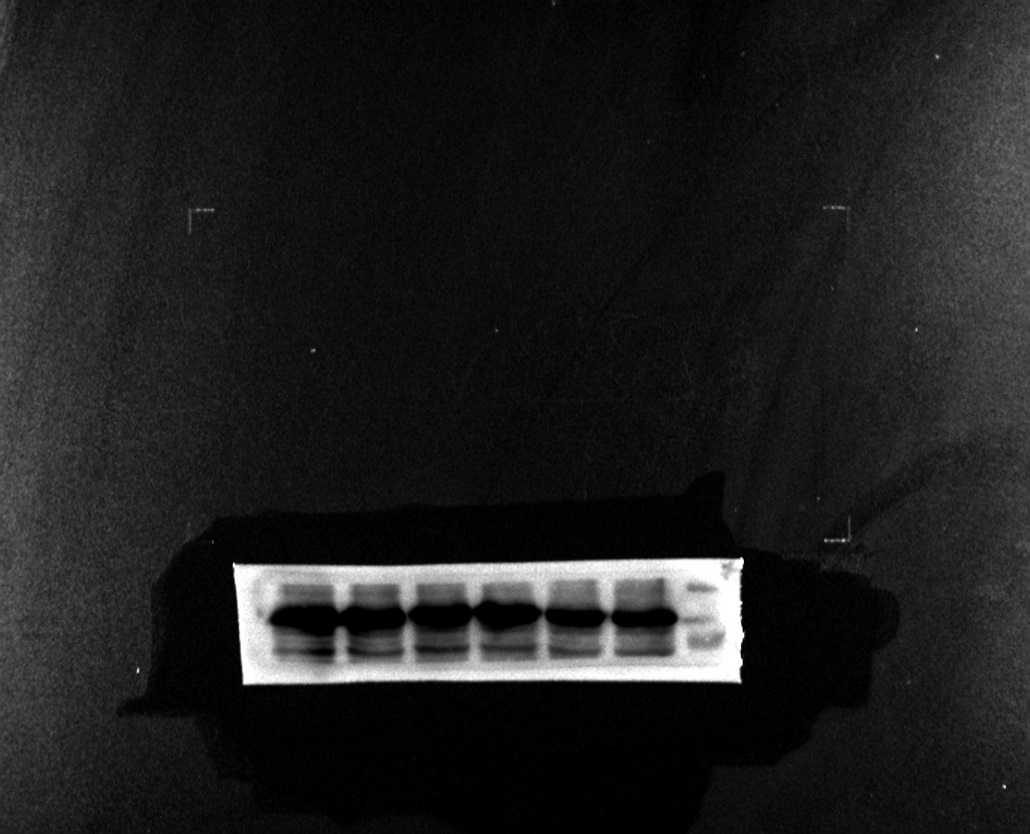


WB: GAPDH (red box)

**Fig 5D Uncropped blot**

Maker


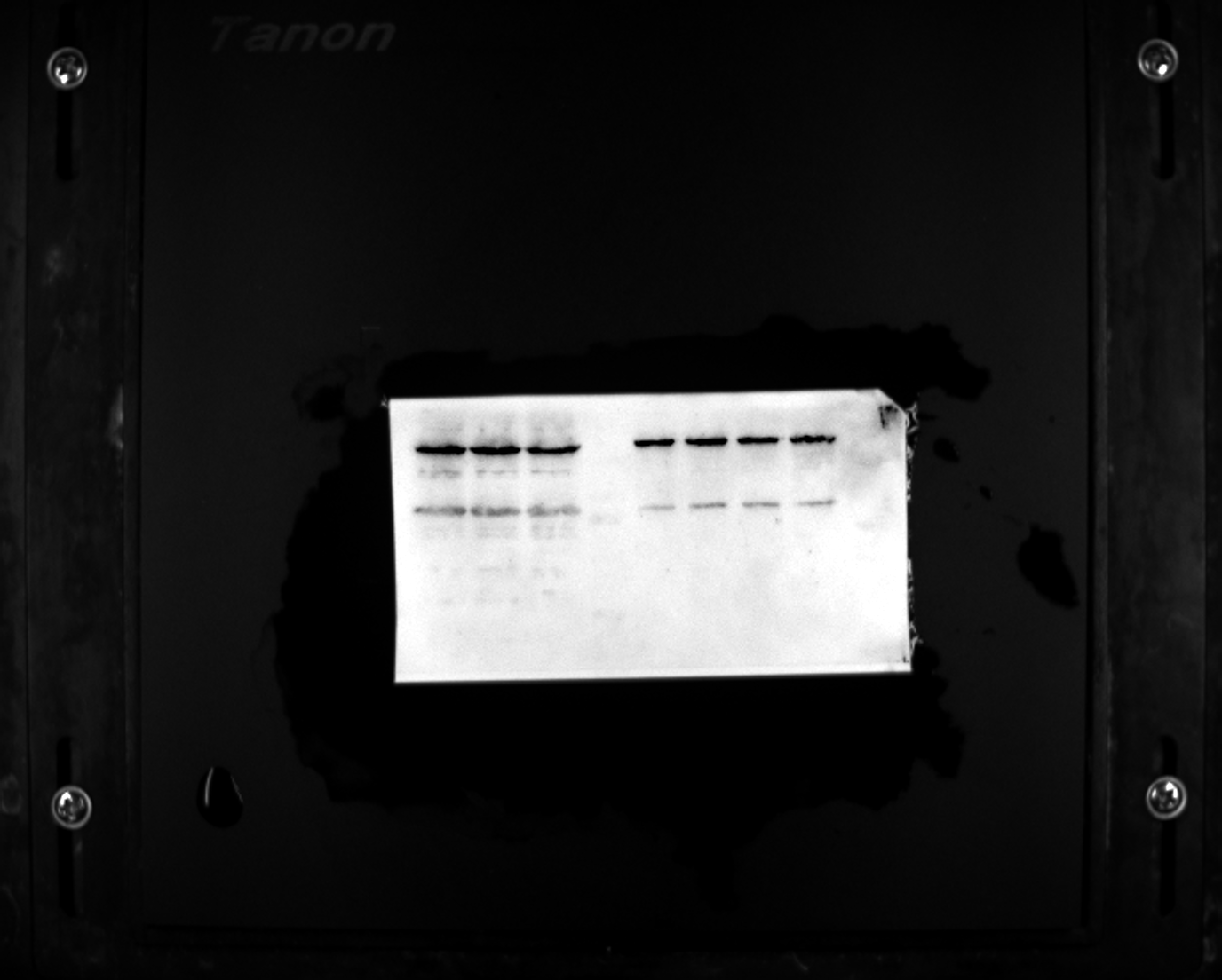


WB: VIM

WB: GAPDH

**Fig 6A Uncropped blot**

Maker


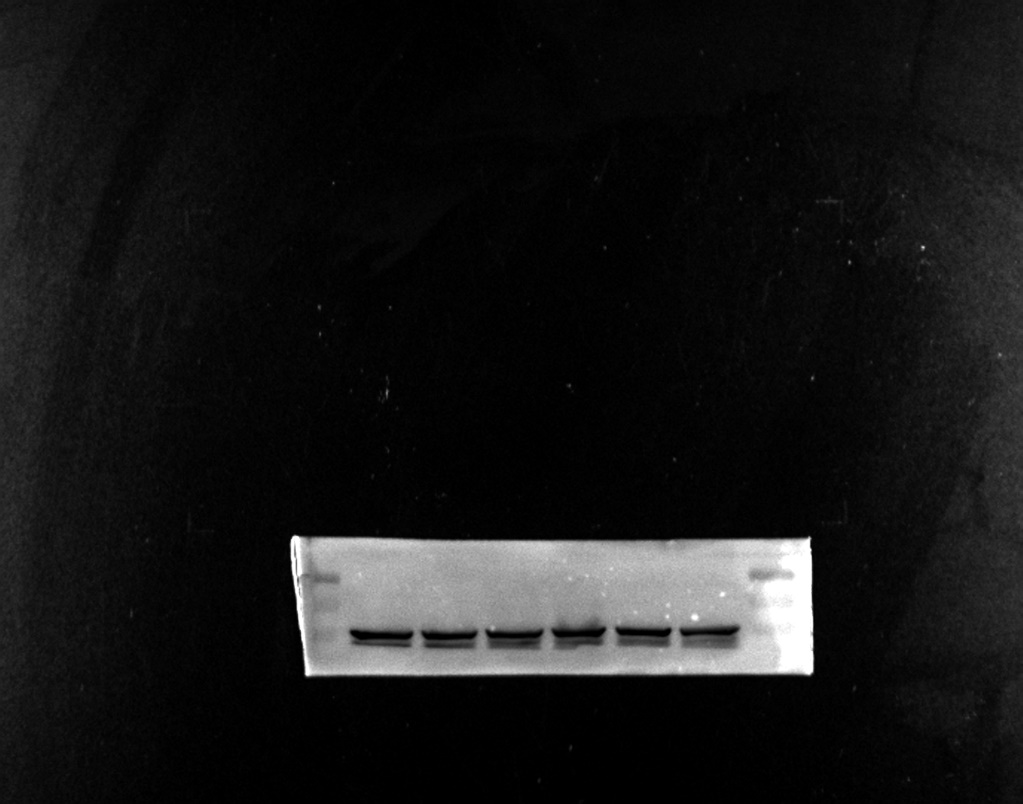


WB: VIM (red box)

WB: RPSA


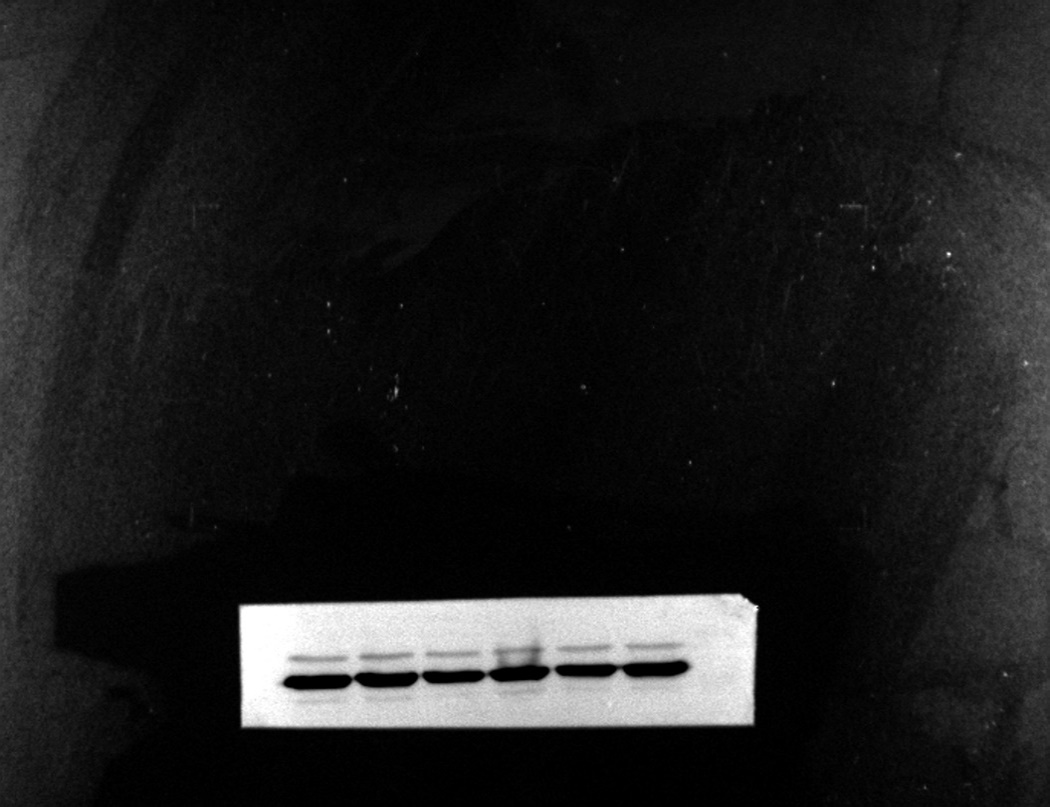


WB: GAPDH (red box)

**Fig 6B Uncropped blot**


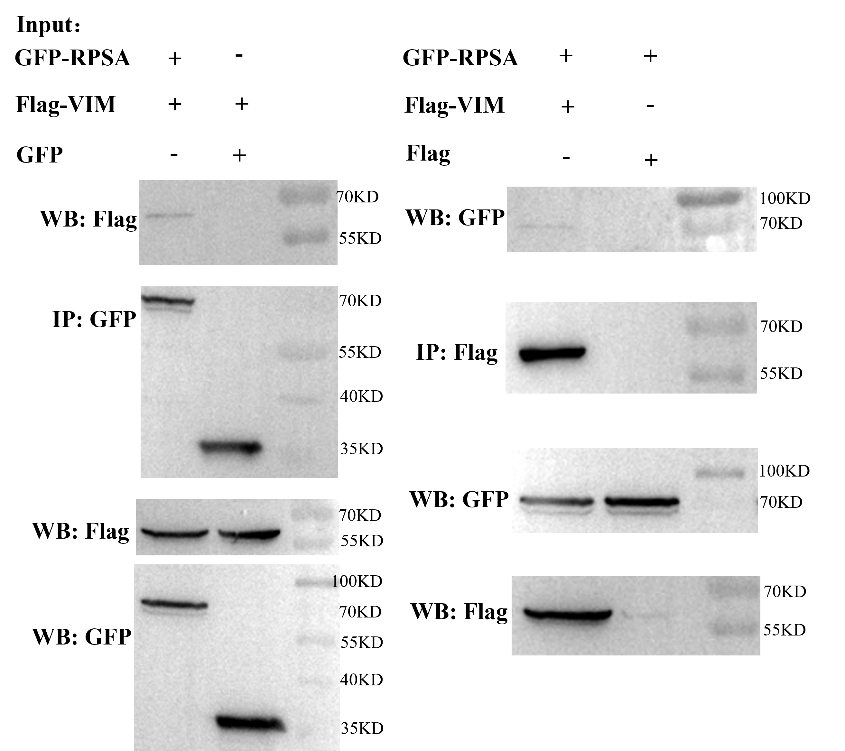


8

7

6

5

4

1

2

3

**Fig 6D**


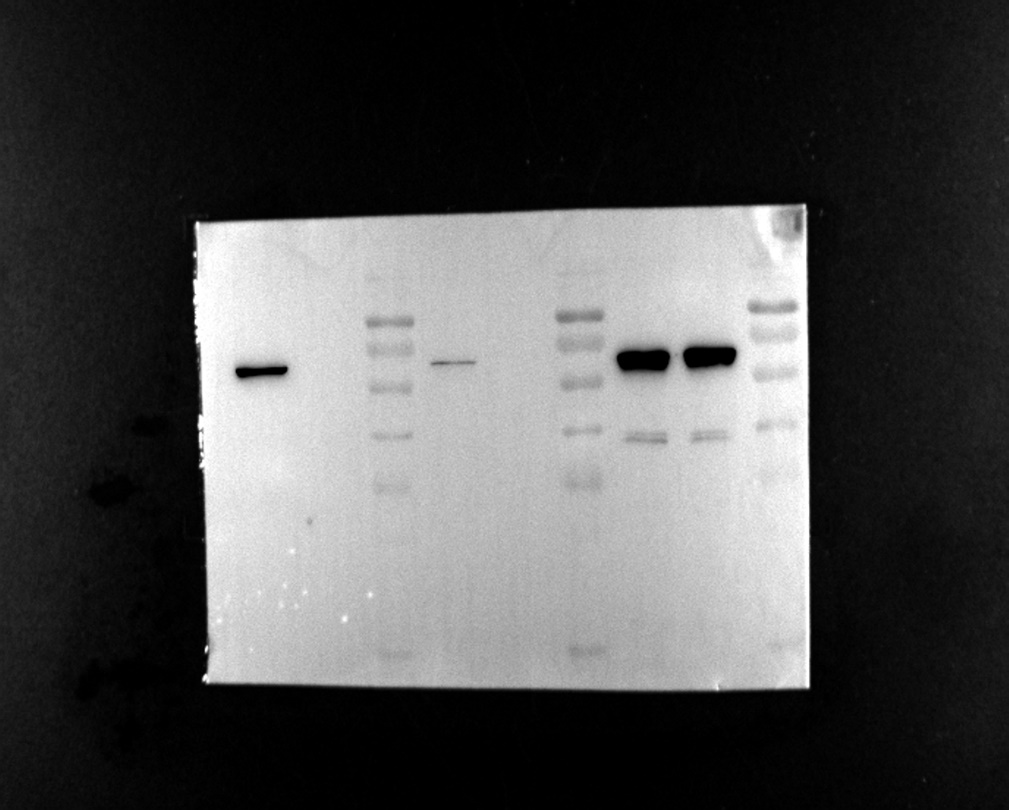


6

1


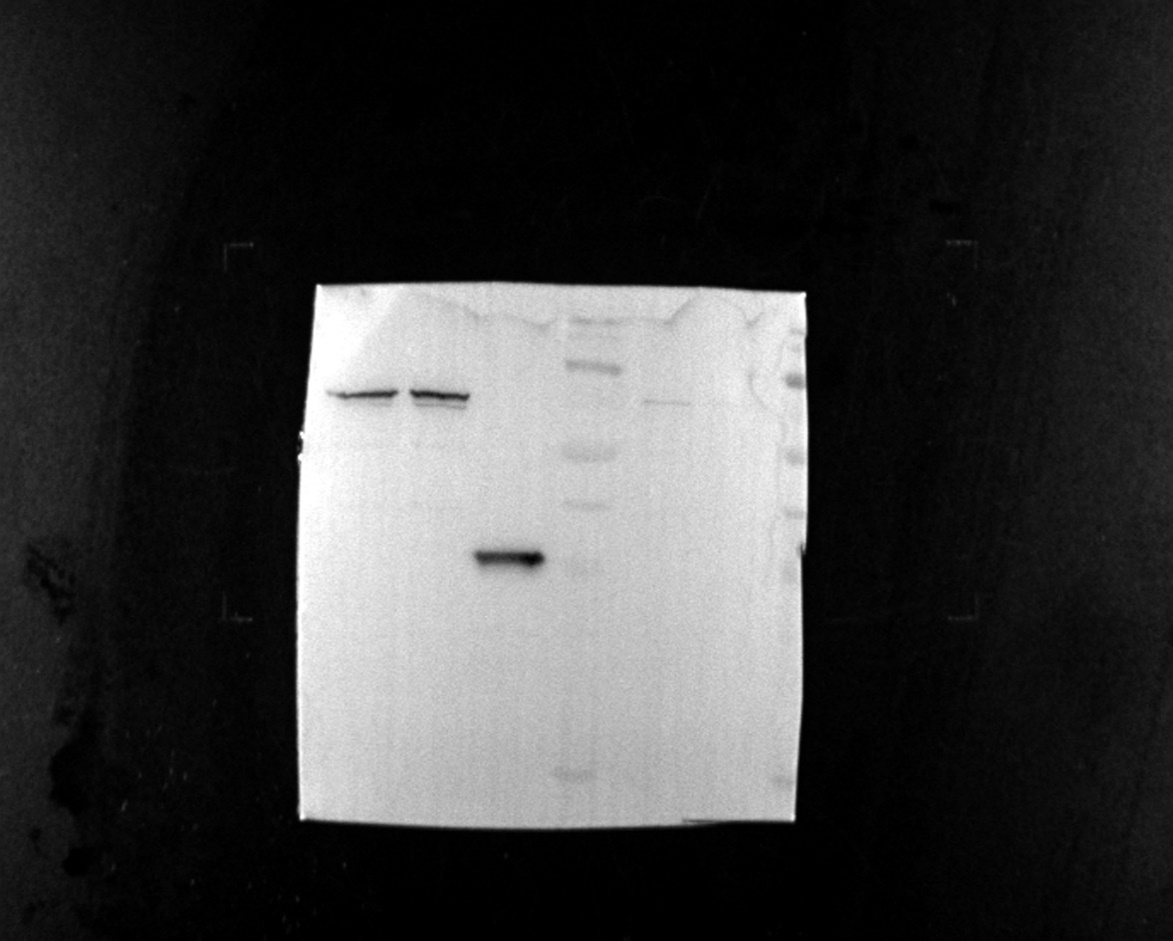


2

5


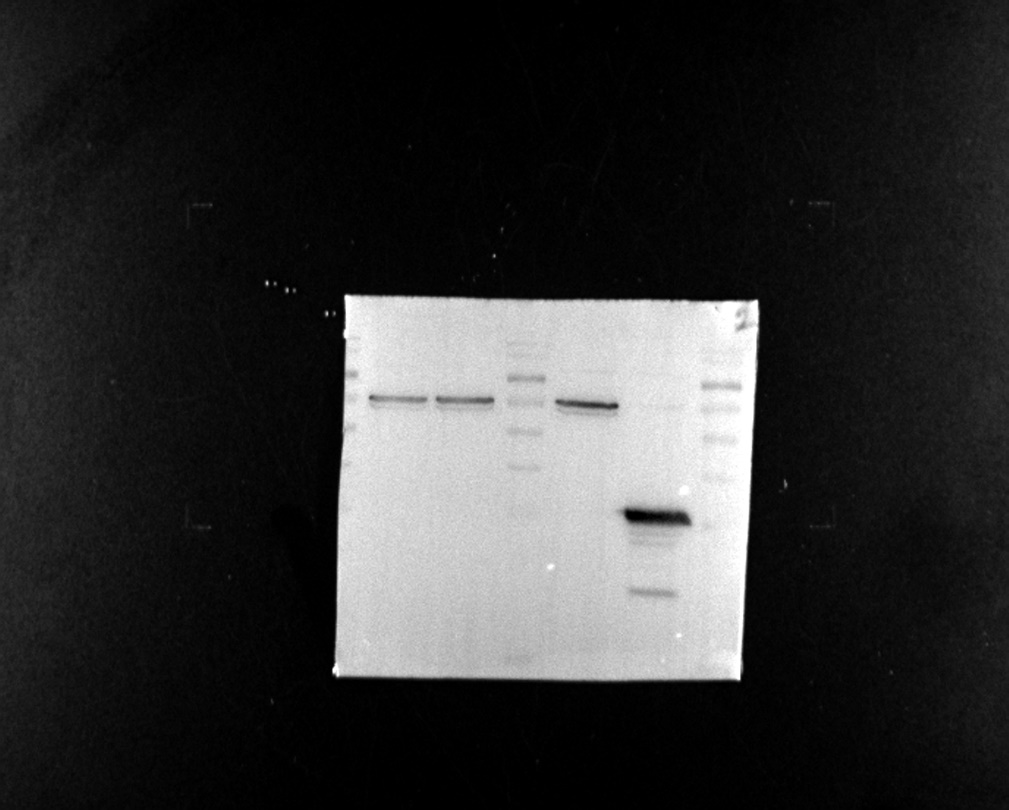


4

7


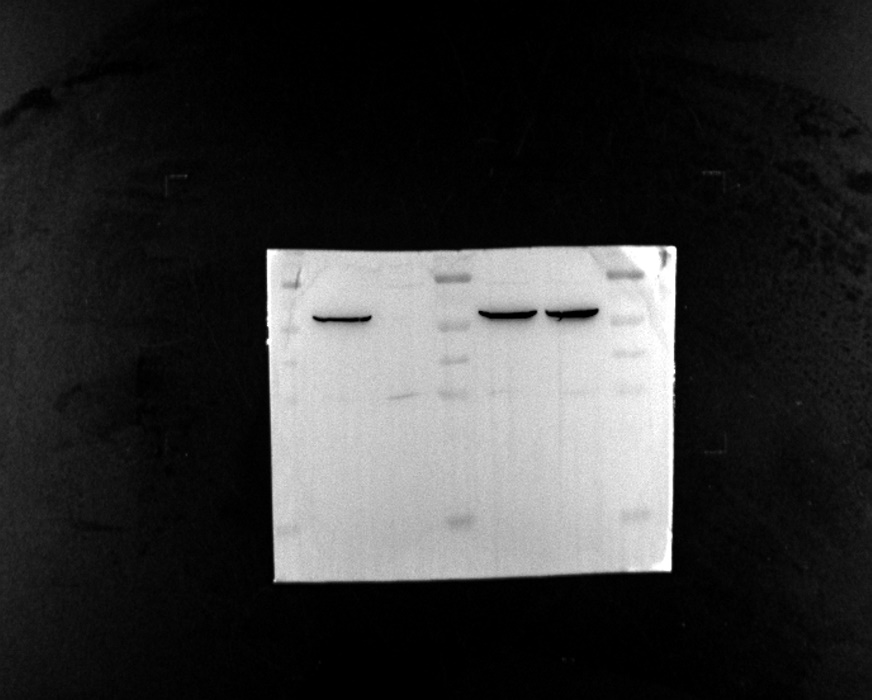


3

8

**Fig 6D Uncropped blot (The Uncropped blot image corresponds to Figure 6D by number)**

Maker


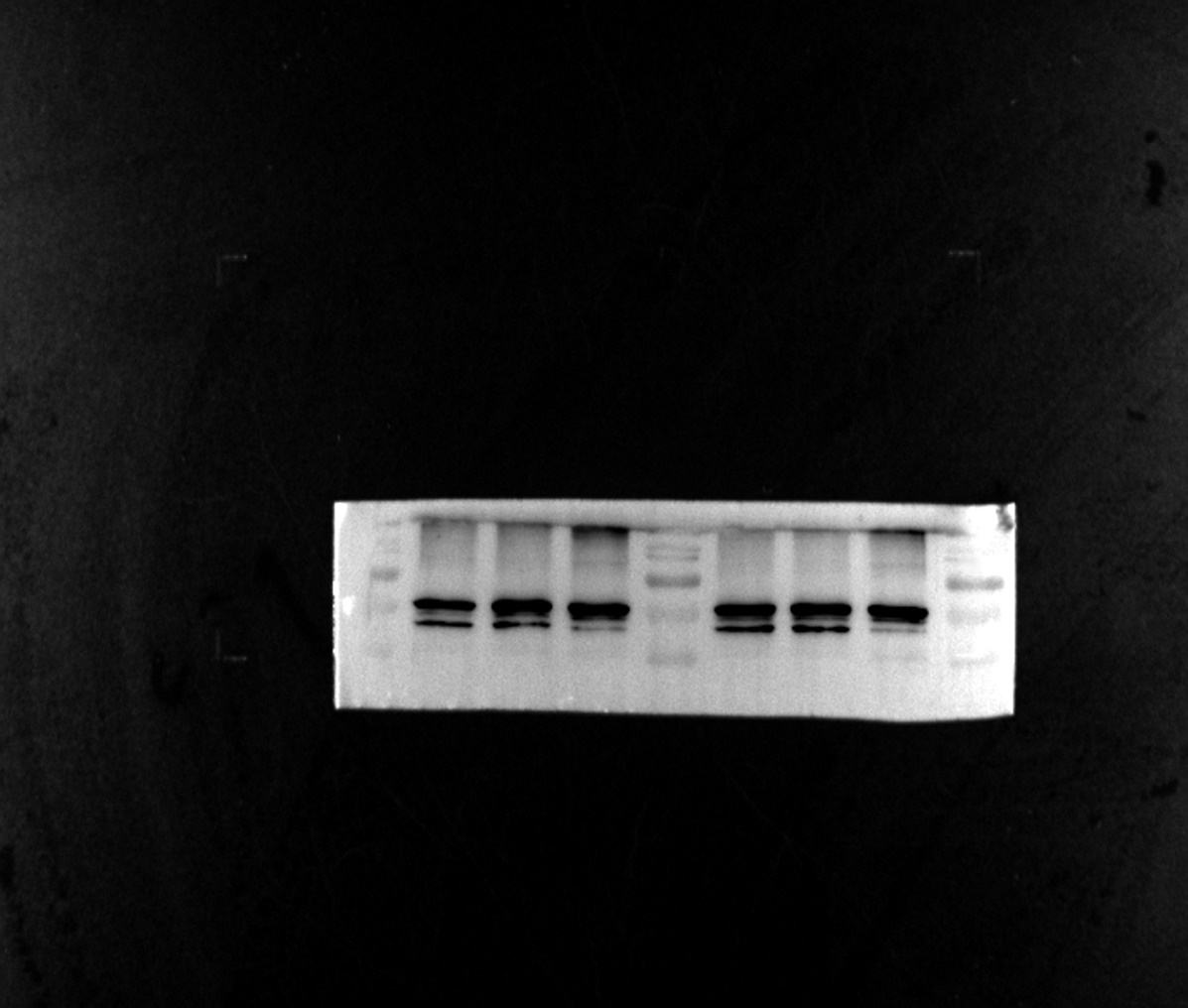


**Fig 6E Uncropped blot**

Maker


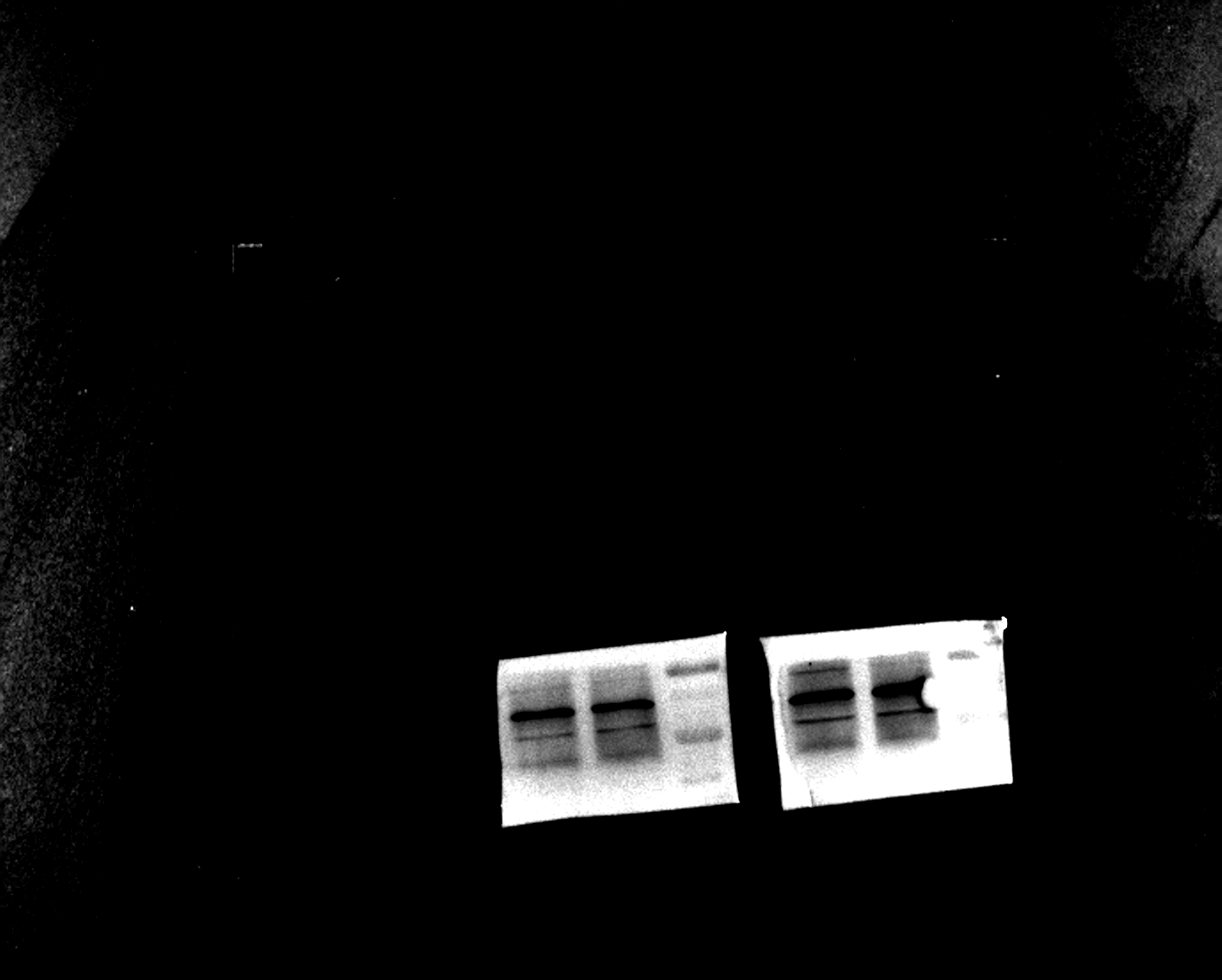


**Fig 6F Uncropped blot**

Maker


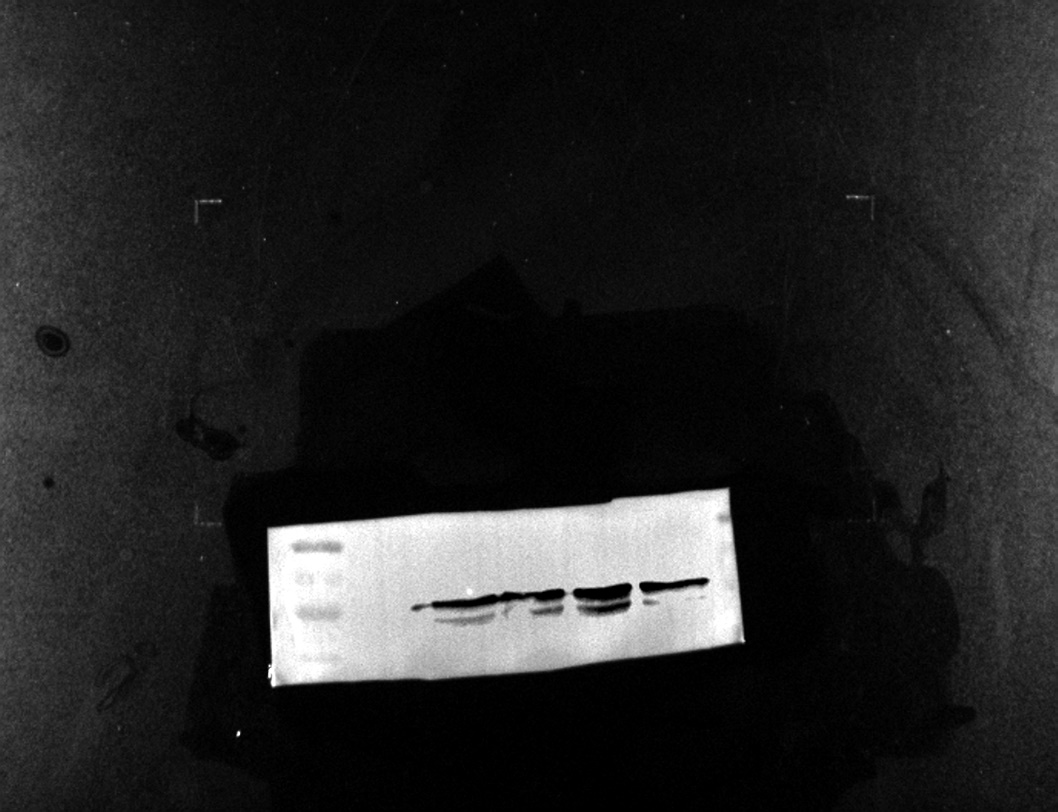


WB: His


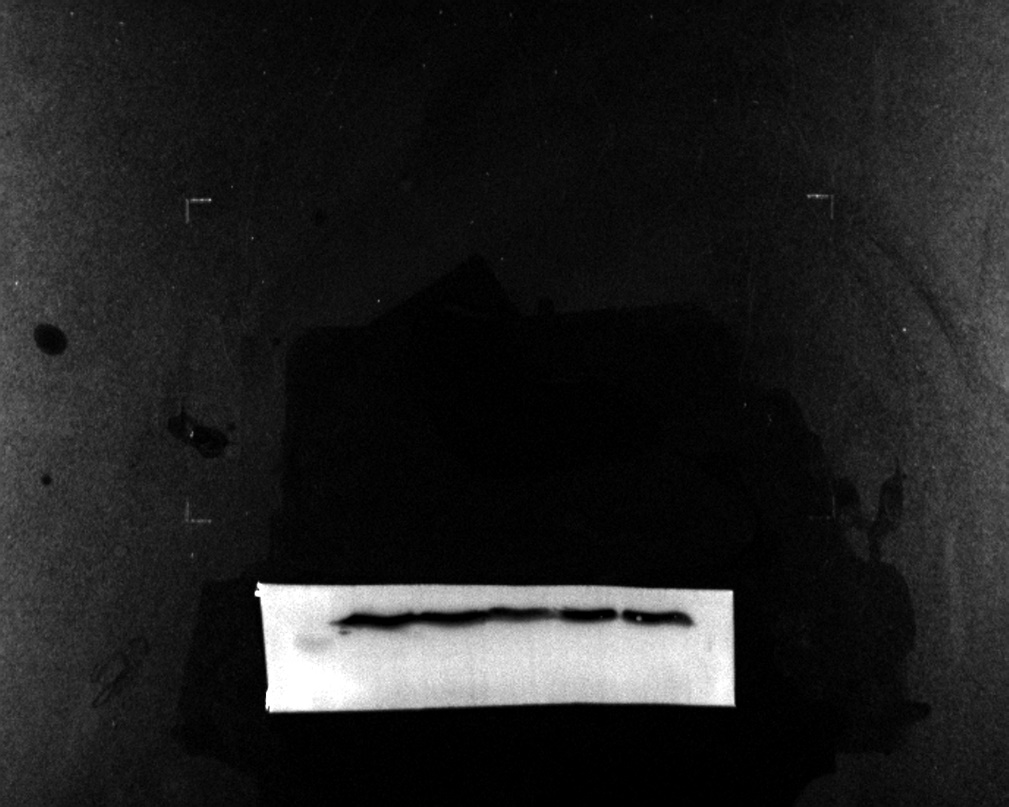


WB: GAPDH

**Fig 8D Uncropped blot**
